# Synthesis and Electrochromism of Highly Organosoluble Polyamides and Polyimides with Bulky Trityl-Substituted Triphenylamine Units

**DOI:** 10.3390/polym9100511

**Published:** 2017-10-14

**Authors:** Sheng-Huei Hsiao, Wei-Kai Liao, Guey-Sheng Liou

**Affiliations:** 1 Department of Chemical Engineering and Biotechnology, National Taipei University of Technology, No. 1, Sec. 3, Chunghsiao East Rd., Taipei 10608, Taiwan; s880497@gmail.com; 2 Institute of Polymer Science and Engineering, National Taiwan University, No. 1, Sec. 4, Roosevelt Rd., Taipei 10617, Taiwan

**Keywords:** trityl (triphenylmethyl), triphenylamine, polyamides, polyimides, redox-active polymers, electrochromic polymers

## Abstract

Two series of polyamides and polyimides containing bulky trityl-substituted triphenylamine units were synthesized from condensation reactions of 4,4′-diamino-4′′-trityltriphenylamine with various dicarboxylic acids and tetracarboxylic dianhydrides, respectively. The polymers showed good solubility and film-forming ability. Flexible or robust films could be readily obtained via solution-casting. The use of aliphatic diacid or dianhydride reduces interchain charge transfer complexing and leads to colorless polyamide and polyimide films. These polymers showed glass-transition temperatures in the range of 206–336 °C. Cyclic voltammograms of the polyamide and polyimide films displayed reversible electrochemical oxidation processes in the range of 0–1.0 or 0–1.3 V. Upon oxidation, the color of polymer films changes from colorless to blue-green or blue. As compared to the polyimide counterparts, the polyamides showed lower oxidation potentials and thus a higher electrochromic stability and coloration efficiency. Simple electrochromic devices were also fabricated as a preliminary investigation for electrochromic applications of the prepared polymers.

## 1. Introduction

Aromatic polyamides and polyimides are classified as high performance polymers by their outstanding elevated thermal stability, high strength and stiffness, and broad chemical resistance [1,2,3,4,5,6,7,8]. They have been widely used in the advancement of technologies in many fields, such as automotive, aerospace, sport and safety equipment, and electronics. However, the fabrication of aromatic polyamides and polyimides, through the melt or the solution processing, is generally difficult because they have high softening or melting temperatures and are insoluble in most organic solvents. Fabrication of aramid fibers was generally carried out from anisotropic solutions in strong acids, such as concentrated sulfuric acid or in polar organic solvents containing dissolved inorganic salts. Although aromatic polyimides can be processed through a two-step process based on soluble poly(amic acid) precursors, this common technique still retains some inherent problems, such as instability of poly(amic acid)s and release of water during thermal curing. Solubility improvements of aromatic polyamides and polyimides could be achieved by macromolecular engineering, mainly by designing and synthesizing new monomers [3,9,10]. Moreover, most aromatic polyimide films usually exhibit deep color due to charge transfer complexing (CTC) [11,12]. Organosoluble and colorless aromatic polyimides could be obtained from the monomer combination of less electron-accepting bis(ether anhydride)s and less electron-donating trifluoromethyl-substituted diamines [13,14]. The use of aliphatic dianhydride and/or diamine monomers also could decrease the CTC effect and led to low color or colorless polyimides [15,16].

Triarylamine-based small molecules, dendrimers, star compounds, or polymers exhibit attractive hole-transporting properties and are promising candidates to be amenable for use in a variety of optoelectronic devices [17,18,19,20,21,22]. In addition, triarylamine-containing polymers have been reported to exhibit reversible electrochemical processes and stable electrochromic properties [23,24,25]. We disclosed in 2005 that aromatic polyimides bearing triphenylamine units show interesting redox-active and electrochromic behaviors in addition to their inherent properties, such as high thermal stability [26]. Since then, a lot of condensation-type triarylamine-based high performance polymers, typified by aromatic polyimides [27,28,29,30,31,32] and polyamides [33,34,35,36,37,38], have been synthesized and investigated for electrochromic applications. A comprehensive review on aromatic polyamides and polyimides containing triarylamine moieties for electrochromic applications has been reported by Yen and Liou [39]. Due to the presence of packing-disruptive, three-dimensional triarylamine moieties, these polymers, especially for polyamides, are generally organosoluble and can be easily fabricated into amorphous thin film via solution-casting process. This is helpful for their applications in optoelectronic devices.

Incorporating bulky three-dimensional groups into the backbones of aromatic polyimides and polyamides might enhance the solubility without much compromising their thermal and mechanical properties. For example, Zhang et al. introduced a non-polar bulky triphenylmethyl (trityl) pendent group onto polyimides, which exhibited a high solubility and low dielectric constant [40]. Aromatic polyimides and polyamides with double propeller-shaped trityl-substituted triphenylamine units have been reported by Zhang’s group [41] and Banerjee’s group [42], respectively. The obtained polyimides and polyamides exhibited good solubility in many aprotic solvents and had moderate to high glass transition temperatures. Moreover, the incorporation of bulky trityl-substituted triphenylamine units increases the interchain spacing and reduces the packing efficiency, thereby increasing the intrinsic microporosity. Thus, they also exhibited a good gas separation performance. Although the gas transport properties of polyimides and polyamides bearing trityl-substituted triphenylamine units have been reported, little information was known for their electrochemical and electrochromic properties. As a continuation of our devotion to research and developments in easily processable high performance polymers with electrochromic function, herein we synthesized and characterized some aromatic and semi-aromatic polyamides and polyimides containing trityl-substituted triphenylamine units, and the electrochemistry, spectroscopy, and electrochromic cycling stability of the polymer films were also investigated. In addition to increased solubility caused by the incorporation of twisted nonplanar trityl substituent and triphenylamine group, the increased free volume was expected to allow better charge transfer at doping, and thus leading to fast switch speeds.

## 2. Experimental Section

### 2.1. Materials

Triphenylmethyl chloride (Acros, Geel, Belgium), aniline (Acros), cesium fluoride (CsF, Acros), 10% palladium on activated carbon (Pd/C, Fluka, Milwaukee, WI, USA), triphenyl phosphite (TPP, Acros), and pyridine (Py, wako, San Francisco, CA, USA) were used as received. *N*-Methyl-2-pyrrolidine (NMP, TEDIA, Fairfield, OH, USA) and *N*,*N*-dimethylacetamide (DMAc, TEDIA) were distilled over calcium hydride. Terephthalic acid (**4a**) (Acros), 4,4′-dicarboxydiphenyl ether (**4b**) (TCI, Tokyo, Japan), 2,2′-bis(4-carboxyphenyl)hexafluoropropane (**4c**) (TCI), adipic acid (**4d**) (Acros), and 1,4-cyclohexanedicarboxylic acid (**4e**) (Acros) were thoroughly dried by heating at 150 °C under vacuum for 10 h before use. 4,4′-Oxydiphthalic dianhydride (ODPA; **6a**) (Oxychem, Dallas, TX, USA), 4,4′-hexafluoroisopropylidenediphthalic dianhydride (6FDA; **6b**) (Hoechst Celanese, Irving, TX, USA), and 1,2,4,5-cyclohexanetetracarboxylic dianhydride (HPMDA; **6c**) (from Changzhou Sunlight Pharmaceutial Co., Changzhou, China) were purified by sublimation under vacuum. Tetrabutylammonium perchlorate (TBAP; Bu_4_NClO_4_) (TCI) was purified by recrystallization from ethyl acetate. Heptyl viologen tetrafluoroborate (HV(BF_4_)_2_) was synthesized according to the procedure described in literature [43].

### 2.2. Monomer Synthesis

**Figure 1:**
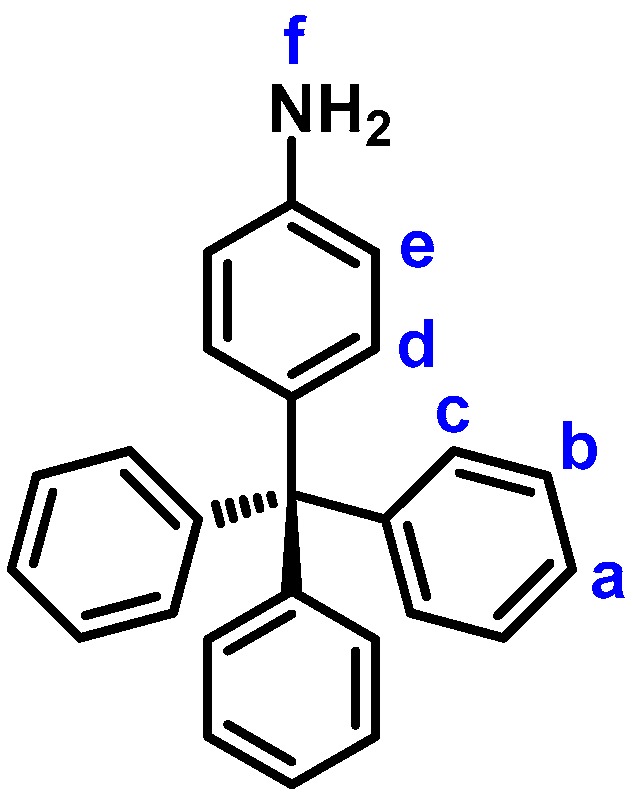
Structure and codes of hydrogen atoms of 4-trylaniline.

**4-Tritylaniline (1).** In a 250 mL three-neck flask equipped with a stirring bar and nitrogen gas inlet were placed 14.4 g (50 mmol) of triphenylmethyl chloride and 12.5 mL of aniline. The reaction mixture was heated with stirring at reflux temperature for 10 min under nitrogen atmosphere, and then the solution was slowly cooled to 95 °C. Then, 240 mL of 2.0 M solution of hydrochloric acid and 200 mL of methanol were added into the flask. After refluxing for another 30 min, the reaction mixture was cooled to the room temperature. The precipitated grey product was collected by filtration and dried to give 15.1 g (90% yield) of the desired compound **1** as a grey powder; m.p. = 257−258 °C, measured by DSC at 10 °C min^−1^. IR (KBr): 3474, 3380 cm^−1^ (–NH_2_ stretch). ^1^H NMR (600 MHz, DMSO-*d*_6_, δ, ppm) (for the peak assignments, see Figure 1): 4.99 (s, 2H, H_f_), 6.47 (d, *J* = 8.7 Hz, 2H, H_e_), 6.75 (d, *J* = 8.7 Hz, 2H, H_d_), 7.12 (d, *J* = 8.3 Hz, 6H, H_c_), 7.17 (t, *J* = 7.3 Hz, 3H, H_a_), 7.26 (t, *J* = 7.7 Hz, 6H, H_b_).

**Figure 2:**
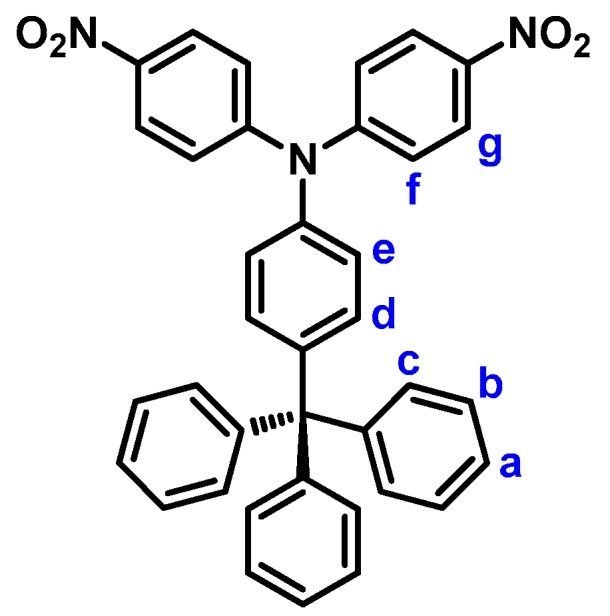
Structure and codes of hydrogen atoms of compound **2**.

**4,4′-Dinitro-4′′-trityltriphenylamine (2)**. Into a 250-mL round-bottom flask equipped with a stirring bar were charged with 6.71 g (20 mmol) of 4-tritylaniline (**1**), 6.77 g (48 mmol) of *p*-fluoronitrobenzene, 7.29 g of cesium fluoride (CsF), and 50 mL of dimethyl sulfoxide (DMSO). The mixture was heated at 140 °C for about 48 h. After cooling, the mixture was poured into 300 mL of methanol, and the precipitated product was collected by filtration and dried to give 8.66 g (75% yield) of the desired dinitro compound **2** as yellow crystals; m.p. = 289−290 °C, measured by DSC at 10 °C min^−1^. IR (KBr): 1584, 1338 cm^−1^ (–NO_2_ stretch). ^1^H NMR (600 MHz, CDCl_3_, δ, ppm) (for the peak assignments, see Figure 2): 7.18–7.25 (m, 17H, H_a_ + H_c_ + H_d_ + H_e_ + H_f_), 7.33 (t, *J* = 7.7 Hz, 6H, H_b_), 8.20 (d, *J* = 9.2 Hz, 4H, H_g_). ESI-MS: calcd. for (C_37_H_27_N_3_O_4_)^+^: *m*/*z* 577.2002; found: *m*/*z* 578.2076 [M + H]^+^. Anal. calcd. for C_37_H_27_N_3_O_4_ (577.64): C, 76.93%; H, 4.71%; N, 7.27%. Found: C, 76.04%; H, 4.80%; N, 7.56%.

**Figure 3:**
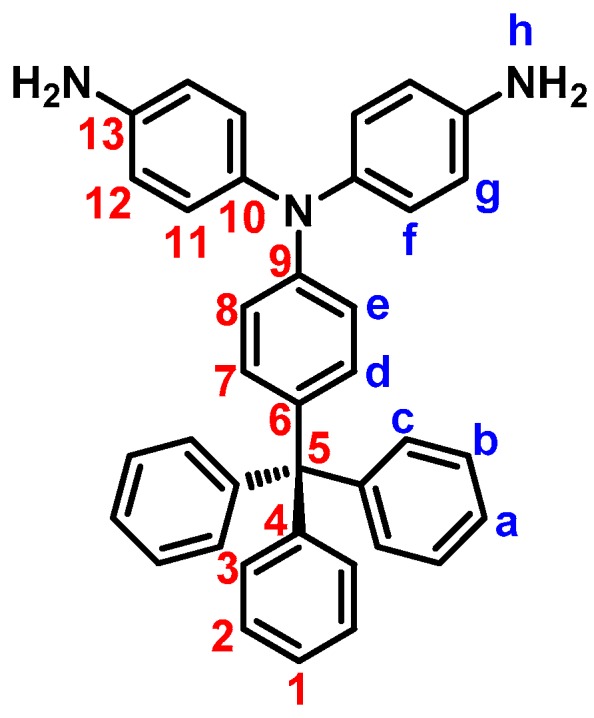
Structure and codes of hydrogen atoms of compound **3**.

**4,4’-Diamino-4”-trityltriphenylamine (3)**. A mixture of 1.5 g (2.6 mmol) of dinitro compound **2,** 2 mL of hydrazine monohydrate, and 0.05 g of 10% Pd/C in 200 mL of ethanol was heated to reflux and held at the reflux temperature for 24 h. The solution was filtered while hot to remove Pd/C, and the filtrate was concentrated under nitrogen atmosphere. After cooling, the precipitated crystals were collected by filtration and dried to give 1.09 g (81% in yield) of pure diamino compound **3**; m.p. = 244−246 °C (by DSC). IR (KBr): 3342, 3208 cm^−1^ (–NH_2_ stretch). ^1^H NMR (600 MHz, DMSO-*d*_6_, δ, ppm) (for the peak assignments, see Figure 3): 4.95 (s, 4H, H_h_), 6.46 (d, *J* = 9.0 Hz, 2H, H_e_), 6.52 (d, *J* = 8.7 Hz, 4H, H_g_), 6.81 (d, *J* = 9.0 Hz, 2H, H_d_), 6.82 (d, *J* = 8.7 Hz, 4H, H_f_), 7.13 (d, *J* = 7.4 Hz, 6H, H_c_), 7.17 (t, *J* = 7.0 Hz, 3H, H_a_), 7.27 (t, *J* = 7.7 Hz, 6H, H_b_). ^13^C NMR (150 MHz, DMSO-*d*_6_, δ, ppm): 63.6 (C^5^), 114.6 (C^8^), 114.7 (C^12^), 125.6 (C^1^), 127.4 (C^2^), 127.6 (C^11^), 130.3 (C^3^), 130.7 (C^7^), 134.8 (C^6^), 135.5 (C^10^), 145.7 (C^13^), 146.8 (C^4^), 147.2 (C^9^). ESI–MS: calcd. for (C_37_H_27_N_3_O_4_)^+^: *m*/*z* 517.2518; found: *m*/*z* 518.2596 [M + H]^+^. Anal. calcd. for C_37_H_31_N_3_ (517.67): C, 85.85%; H, 6.04%; N, 8.12%. Found: C, 85.69%; H, 6.41%; N, 7.70%.

### 2.3. Synthesis of Polyamides

**Figure 4:**
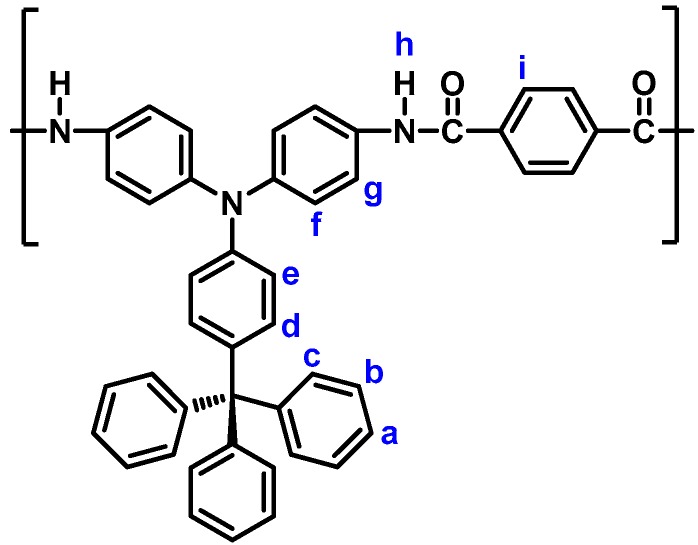
Structure and codes of hydrogen atoms of polyamide **5a**.

The phosphorylation polyamidation technique was used to prepare the polyamides. As a representative example, the synthetic procedure of polyamide (PA) **5a** is described as follows. A solution of diamine monomer **3** (0.5176 g; 1 mmol), of terephthalic acid (**4a**) (0.1661 g; 1 mmol), dried calcium chloride (0.1 g), and TPP (1.0 mL) in pyridine (0.3 mL) and NMP (1.0 mL) was heated at 120 °C for 3 h. A pale yellow fibrous precipitate was obtained when pouring the polymer solution into methanol. The yield of PA **5a** is almost quantitative. The inherent viscosity of PA **5a** was 0.49 dL/g, in DMAc on 0.5 g dL^−1^ at 30 °C. IR (film): 3300 cm^−1^ (N−H stretch), 1660 cm^−1^ (C=O stretch). ^1^H NMR (600 MHz, DMSO-*d*_6_, δ, ppm) (Figure 4): 6.88 (d, 2H, H_e_), 7.03 (d, 2H, H_d_), 7.06 (d, 4H, H_f_), 7.17−7.20 (m, 9H, H_a_ + H_c_), 7.30 (t, 6H, H_b_), 7.76 (d, 4H, H_g_), 8.07 (s, 4H, H_i_), 10.37 (d, 2H, H_h_).

### 2.4. Synthesis of Polyimides

Polyimides (PIs) **7a** and **7c** were synthesized by the one-step method. A 50-mL two-necked flask equipped with a magnetic stirrer, as well as a nitrogen inlet and outlet, was charged with equimolar amounts of each monomer (1.0 mmol of dianhydride ODPA or HPMDA and 1.0 mmol of diamine **3**) and 10 mL of *m*-cresol. The reaction mixture was stirred at room temperature for 1 h until complete dissolution of the monomers, and then heated to 80 °C for 4 h. After a few drops of isoquinoline were added, the mixture was heated at 200 °C for 12 h. Water vapor formed during the imidization was continuously removed by a steady flow of nitrogen. After cooling, the resulting solution was poured into 100 mL of methanol. The precipitated polymer was collected by filtration, washed thoroughly with methanol, and dried.

**Figure 5:**
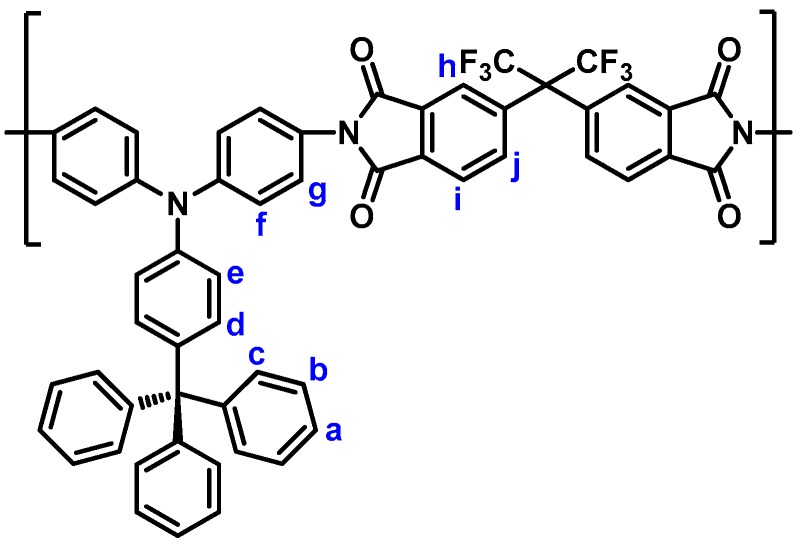
Structure and codes of hydrogen atoms of polyimide **7b**.

PI **7b** was prepared from 6FDA and diamine **3** via the two-step chemical imidization method. The diamine monomer **3** (0.518 g 1.0 mmol) was dissolved in CaH_2_-dried DMAc (4.1 mL) in a 50-mL round bottom flask. Then, 6FDA (0.444 g 1.0 mmol) was added to the diamine solution in one portion. Thus, the solid content of the solution is approximately 20 wt %. The mixture was stirred at room temperature for about 6 h to yield a viscous poly(amic acid) solution. Then, 0.5 mL of pyridine and 0.5 mL of acetic anhydride were added to the poly(amic acid) solution, and the mixture was heated at 120 °C for 2 h to effect a complete imidization. The obtained polymer was precipitated into methanol, then filtered and dried under vacuum for 24 h. PI **7b** exhibited inherent viscosity of 0.62 dL g^−1^, as measured in DMAc on 0.5 g dL^−1^ at 30 °C. IR (film): 1780 and 1717 cm^−1^ (imide ring C=O stretch). ^1^H NMR (600 MHz, DMSO-*d*_6_, δ, ppm) (for the peak assignments, see Figure 5): 7.00 (d, *J* = 8.8 Hz, 2H, H_e_), 7.10 (d, *J* = 8.8 Hz, 2H, H_d_), 7.13 (d, *J* = 8.4 Hz, 4H, H_f_), 7.15−7.20 (m, 15H, H_a_ + H_b_ + H_c_), 7.24 (d, *J* = 6.8 Hz, 4H, H_g_), 7.78 (d, *J* = 7.9 Hz, 2H, H_j_), 7.88 (s, 2H, H_h_), 7.95 (d, *J* = 8.1 Hz, 2H, H_i_).

### 2.5. Fabrication of the Electrochromic Devices (ECDs)

#### 2.5.1. Electrodes

EC polymer films were prepared by coating 300 μL solution of the polymer (2 mg/mL in DMAc) onto an ITO glass substrate (25 mm × 30 mm × 0.7 mm, 5 Ω/square). The ITO glass used for EC device was cleaned by ultrasonication with water, acetone, and isopropanol each for 15 min. The polymer was drop-coated onto the ITO glass with an active area (20 mm × 20 mm) then dried under vacuum. The thickness of dry polymer film is about 250 nm. The periphery of the coated polymer film was surrounded with a thermally cured epoxy resin adhesive by a full-auto dispenser, and one injection port was left. Next, a blank ITO glass was covered on the polymer-coated ITO glass. The two electrodes were pressed together using a subpress, leaving a 120 μm gap distance (controlled by beaded glasses dispersed in the adhesive). After that, the electrodes were heated at 120 °C for 6 h.

#### 2.5.2. Electrolytes

A highly transparent and conductive semi-gelled electrolyte based on 10 wt % poly(methyl methacrylate) (PMMA) (*M*w: 120,000), and LiBF_4_ was plasticized with propylene carbonate (PC). PMMA (0.78 g) was dissolved in PC (5.5 mL) and LiBF_4_ (0.3 g) was added to the polymer solution as supporting electrolyte. Moreover, the HV-containing electrolyte (0.02 M) in this study was prepared by following procedures: PMMA (0.78 g) was dissolved in PC (5.5 ml), then LiBF_4_ (0.3 g) and HV(BF_4_)_2_ (0.06 g) were added to the polymer solution, and then the mixture was thoroughly mixed to obtain a semi-gelled HV-electrolyte (a gel electrolyte containing HV(BF_4_)_2_).

#### 2.5.3. Assembly

The prepared electrolyte was injected by a subpress through the injection port into the gap between the two electrodes, using a 1 mL syringe. Finally, the injection port of the device was sealed using UV-curable sealant.

## 3. Results and Discussion

### 3.1. Monomer Synthesis

**Scheme 1:**
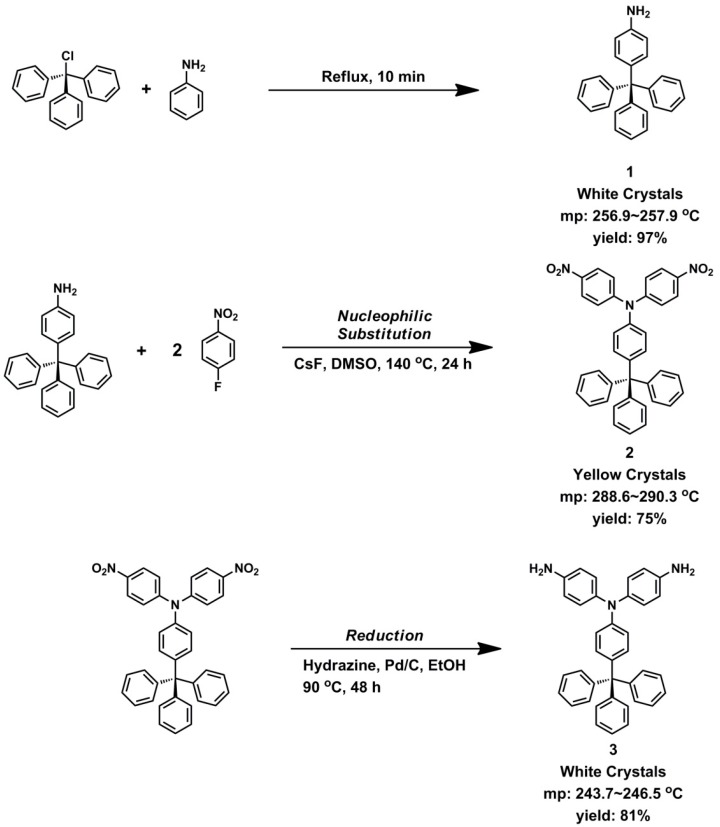
Synthesis of 4,4′-diamino-4′′-trityltriphenylamine (**3**).

**Figure 6:**
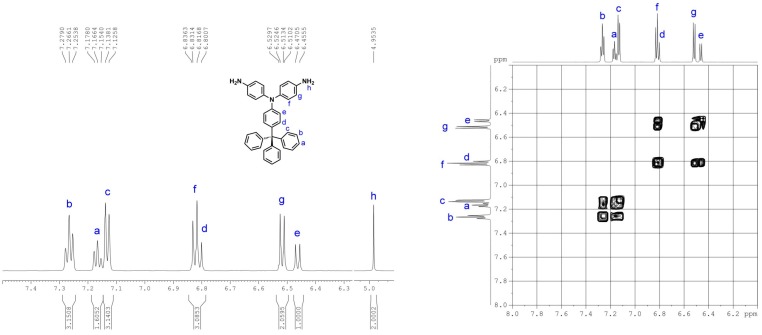
^1^H NMR spectra of 4,4′-diamino-4′′-trityltriphenylamine (**3**), measured in dimethyl sulfoxide (DMSO)-*d*_6_.

**Figure 7:**
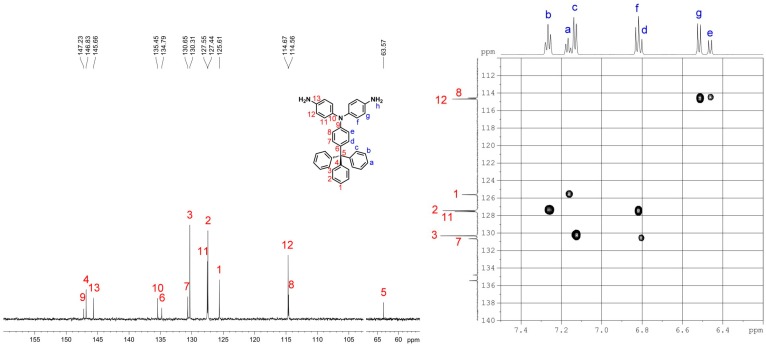
^13^C NMR and C-H HMQC spectra of 4,4′-diamino-4′′-trityltriphenylamine (**3**) in DMSO-*d*_6_.

By applying the literature method [41,42], 4,4′-diamino-4′′-trityltriphenylamine (**3**) was synthesized by a three-step synthetic route (Scheme 1). First, *para*-tritylation of aniline with triphenylmethyl chloride gave 4-tritylaniline (**1**) in a good yield [40,44]. Then, 4,4′-dinitro-4′′-trityltriphenylamine (**2**) was obtained by CsF-mediated fluoro-displacement of *p*-fluoronitrobenzene with 4-tritylaniline in DMSO. Finally, compound **2** was reduced by hydrazine and Pd catalyst in ethanol to **3**. The structures of all the prepared compounds were confirmed by IR and NMR spectroscopies. Figure S1 (Supplementary Materials) illustrates FT-IR spectra of compounds **1**−**3**. All of the nitro and amino functional groups could be evidenced by their characteristic absorptions. The ^1^H NMR spectra of 4-tritylaniline (**1**) and dinitro compound **2** are summarized in Figure S2 (Supplementary Materials). The NMR spectra of the diamine monomer **3** are compiled in Figure 6 and Figure 7. The ^1^H NMR spectrum of **3** is consistent with those reported in literature [41,42]; however, exact assignments of all resonance signals are only shown here. The ^13^C NMR spectrum of diamine monomer **3** and complete assignments of all peaks are first reported in this work (see Figure 7). All of the ^1^H and ^13^C NMR signals are well assigned to the structure of **3**. Mass analysis results of compounds **2** and **3** (Figure S3) are also in good agreement with their calculated values.

### 3.2. Synthesis of Polyamides

**Scheme 2:**
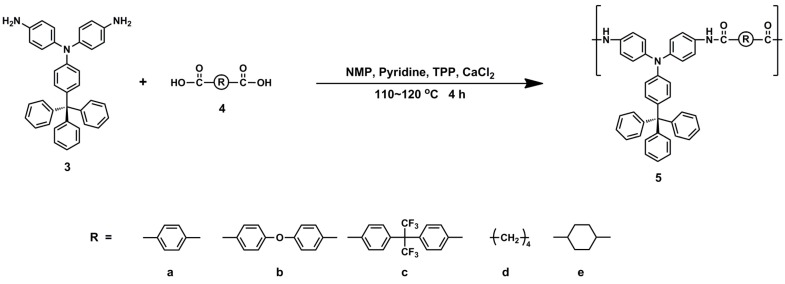

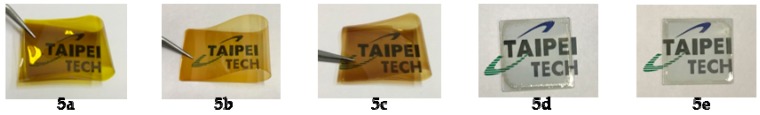
Synthesis of polyamides and their cast films (~25 µm in thickness).

According to the phosphorylation technique described by Yamazaki and co-workers [45], a series of polyamides **5a**–**5e** were synthesized from the diamine monomer **3** with various aromatic or aliphatic dicarboxylic acids (**4a**−**4e**) by means of TPP and pyridine (Scheme 2). As shown in Table 1, the obtained PAs had inherent viscosities in the range of 0.35−0.62 dL g^−1^. Polyamides **5a**–**5c** can be solution-cast into flexible and transparent films (see photos shown in Scheme 2), indicating the formation of high molecular weight polymers. Although **5d** and **5e** have relatively lower inherent viscosities, they can still be casted into robust films on the glass substrates. Their cast films are highly transparent and colorless. The structures of the PAs were confirmed by IR and NMR spectroscopy. Figure S4 (Supplementary Materials) shows the IR spectra of PAs **5a**−**5e**. All of the PAs showed the characteristic absorption bands of the amide group at around 3315 cm^−1^ (N−H stretching) and 1664 cm^−1^ (amide C=O). As a typical example, Figure S5 shows the ^1^H and H−H COSY spectra of polyamide **5a**. All of the resonance signals could be well assigned to the hydrogen atoms in the repeating unit. The resonance peak appearing at 10.37 ppm in the ^1^H NMR spectrum also verifies the formation of amide linkages in the polymer main chain.

### 3.3. Synthesis of Polyimides

**Scheme 3:**
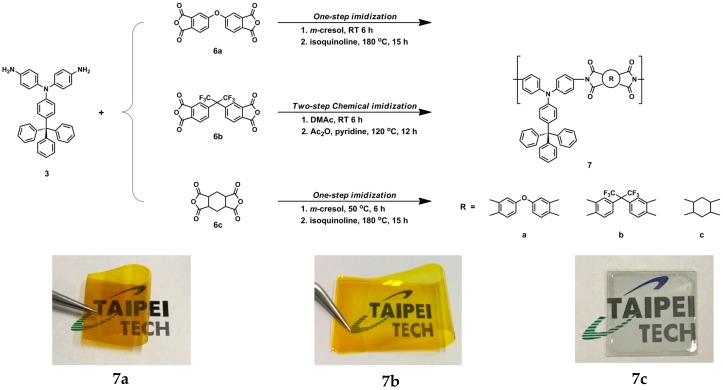
Synthesis of polyimides and their cast films (~25 µm).

The synthetic methods of the PIs **7a**−**7c** are shown in Scheme 3. These PIs were prepared by condensation of diamine compound **3** with aromatic tetracarboxylic dianhydrides ODPA (**6a**), 6FDA (**6b**), and HPMDA (**6c**), respectively. The resulting polyimides exhibited inherent viscosities in the range of 0.25−0.64 dL g^−1^, as listed in Table 1.

The structures of these polyimides were confirmed by IR and NMR analysis. Figure S6 (Supplementary Materials) shows the IR spectra of PIs **7a**−**7c**. Characteristic absorptions at 1780 and 1717 cm^−1^ peculiar to imide groups could be observed in these spectra. Representative ^1^H NMR spectra of polyimide **7b** are presented in Figure S7. The resonance signals of the aromatic protons appear in the range of *δ* 6.9−8.0 ppm. The spectrum agrees well with the repeating unit of PI **7b**.

### 3.4. Properties of Polymers

#### 3.4.1. Solubility

The solubility of the polymers was tested in some common organic solvents at 10% (*w*/*v*). The results are shown in Table 1. Almost all of the polymers are readily soluble in polar solvents that include NMP, DMAc, DMF, DMSO, and *m*-cresol. Except for PAs **5a** and **5b**, all other polymers, are also soluble in less polar THF. All of the polymers exhibit an enhanced solubility in comparison with the analogous triphenylamine-based polymers without the trityl substituent [46]. The enhanced solubility of these polymers can be explained by the introduction of the trityl-substituted triphenylamine unit in the backbones, which hinders with close chain packing and decreases chain-chain interactions. Thus, the high solubility favors their practical applications via solution processing.

#### 3.4.2. Thermal Stability

The thermal properties of all the polymers were studied by DSC and TGA. The relevant data are summarized in Table 2. Typical TGA thermograms of PAs **5c** and **5d** and PIs **7a**−**7c** are reproduced in Figure S8 (Supplementary Materials). The glass-transition temperatures (*T*_g_) of these polymers by DSC were recorded between 206 and 336 °C. Semi-aromatic PA **5d** exhibited relatively lower *T*_g_ values (206 °C) when compared with wholly aromatic **5c** (*T*_g_ = 305 °C) because of the flexible methylene segments. PAs **5a**−**5c** showed slightly higher *T*_g_ values than the corresponding parent triphenylamine-based PAs [46]. This may be a result of decreased freedom of segmental motion caused by the trityl substitution. Aromatic PAs **5a** to **5c** showed no significant weight loss below 400 °C in air or in N_2_. The 10% weight-loss temperatures (*T*_d_^1^^0^) of the PAs in N_2_ and air were recorded in the range of 395–435 °C and 390–460 °C, respectively. As expected, PAs **5d** and **5e** reveal lower decomposition temperatures and char yields than PAs **5a**−**5c** attributable to the less stable aliphatic segments. Because of the rigid imide ring, the PIs exhibited higher *T*_d_ and *T*_g_ values, as compared to the corresponding PAs. The good thermal stability of these PAs and PIs is beneficial to increase the morphological stability and life time of their cast films in device applications.

#### 3.4.3. Electrochemical Property

**Figure 8:**
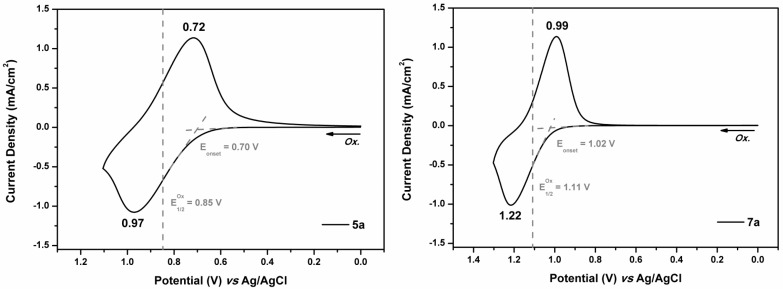
Cyclic voltammogram of the polyamide (PA) **5a** and polyimide (PI) **7a** films on the ITO-coated glass slide in 0.1 M TBAP/MeCN with a scan rate of 50 mV s^−1^.

The electrochemical property of the PAs and PIs was investigated by cyclic voltammetry (CV) with the cast films on ITO-glass in 0.1 M TBAP (Bu_4_NClO_4_)/acetonitrile (MeCN) solution. An Ag/AgCl in saturated KCl solution was used as a reference electrode (calibrated externally using a 5 mM solution of ferrocene/ferrocenium couple). The oxidation half-wave potential (*E*_1/2_^Ox^) of ferrocene was found as 0.44 V vs. Ag/AgCl (see Figure S9, Supplementary Materials). The typical CV diagrams of representative PA **5a** and PI **7a** are depicted in Figure 8, and those of the other polymers are included in the Supplementary Materials (Figures S10 and S11). The oxidation potentials of the polymers are summarized in Table 3. The half-wave potentials (*E*_1/2_) of the polymers were recorded in the ranges of 0.81−0.86 V (for the PAs) and 1.09−1.11 V (for the PIs). It is reasonable that the PIs showed a significant anodic shift of the potential in comparison to their PA counterparts due to the presence of electron-withdrawing imide units. Upon oxidation, the polymer films changed color from colorless to greenish blue or blue. The HOMO (highest occupied molecular orbital) energy levels of the polymers were calculated from cyclic voltammetry and by comparison with ferrocene (5.1 eV) [47]. These data, together with absorption spectra, were then used to obtain the LUMO (lowest unoccupied molecular orbital) energy levels (Table 3).

#### 3.4.4. Electro-Optical Property

**Figure 9:**
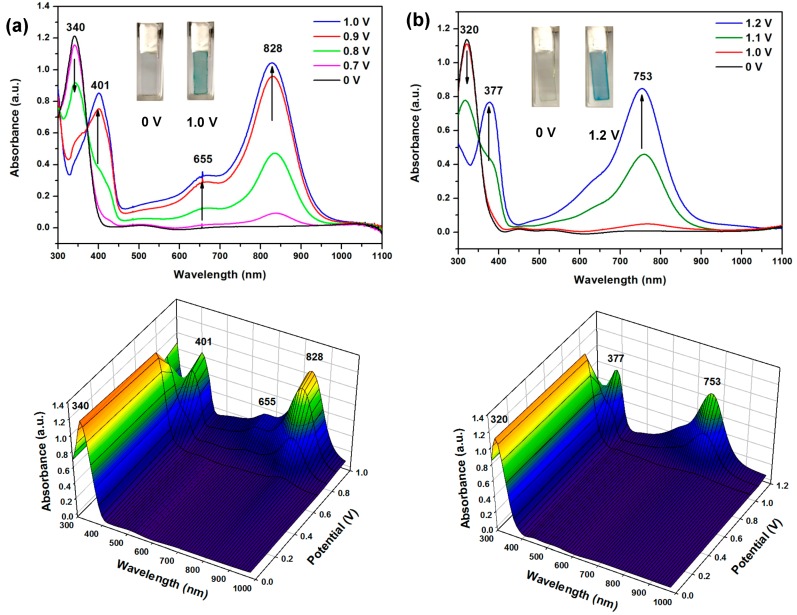
Optical absorption spectra and color changes of the cast films (thickness: 200 ± 30 nm) of (**a**) PA **5b** and (**b**) PI **7a** on the ITO-coated glass slide in 0.1 M TBAP/MeCN at various applied potentials.

In order to measure the optical properties in detail, the changes in the optical absorption profile of the polymer films coated on ITO electrode were recorded in situ to elucidate the electro-optical properties. The UV–vis–NIR absorption spectral change of PA **5b** and PI **7a** films at various applied potentials are illustrated in Figure 9. The spectroelectrographs of other polymers are given in the Supplementary Materials (Figures S12−S16). In the neutral state, the PA films exhibited a strong absorption centered at 320−352 nm, due to the π−π* transitions. Take **5b** as an example. When the applied potential was increased to 1.0 V, the π−π* transition band at 340 nm gradually decreased while new absorption bands at 401, 655, and 828 nm gradually appeared, attributed to the formation of charge carriers. The spectral changes can be easily followed by color change from colorless to greenish blue during doping. Therein, semi-aromatic polymers show a slight blue shift, because the conjugate length is shorter than the wholly aromatic polymer, therefore the color change from greenish blue to blue upon oxidation. PI **7a** showed a similar spectral change upon electro-oxidation; however, the absorption maxima at its doped state exhibited a slight blue shift as compared to PA **5b**.

#### 3.4.5. Electrochromic Switching and Stability

**Figure 10:**
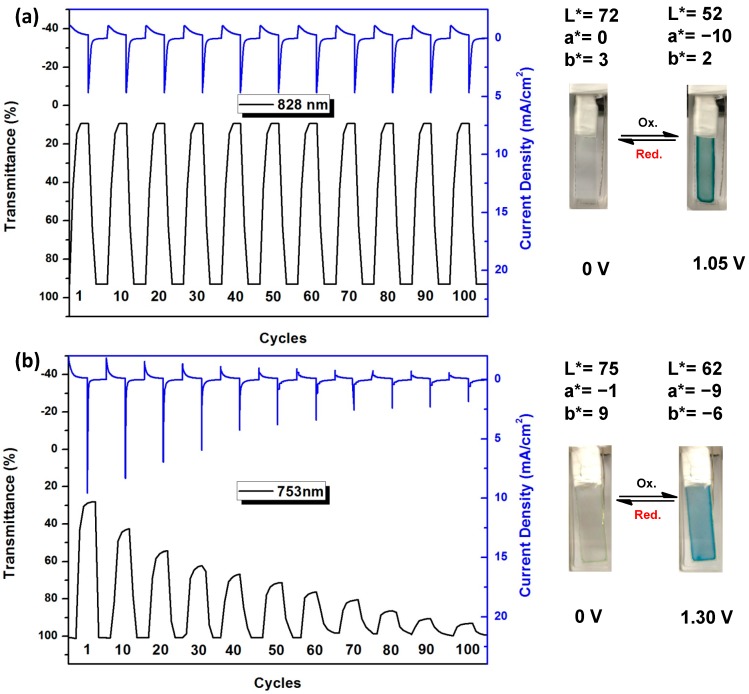
Cheonoabsorptometry experiments for polymer films on the ITO-glass slide (active area: 1 cm^2^, thickness: 200 ± 30 nm): (**a**) PA **5b** switched between 0.0 and 1.05 V with a pulse width of 10 s; (**b**) PI **7a** switched between 0.0 and 1.30 V with a pulse width of 20 s.

In order to determine the switching stability and response time, a square-wave potential method was used and then 0 V and 1.0 V, or 1.3 V was applied for a pulse width of 10 s. While the external applied potentials were switched, the absorbance at selected wavelengths was monitored. Figure 10 shows the percentage transmittance change of PA **5b** film at 828 and PI **7a** film at 753 nm upon potential switching. The decay in electrochromic coloration efficiency (CE = ΔOD/Q_d_) after various switching steps for PA **5b** is shown in Table 4. PA **5b** exhibited good coloration efficiencies of up to 186 cm^2^ C^−1^ at 828 nm at the first switching cycle. It did not lose the electrochromic activity significantly and retained about 95% of its optical response after 100 switching cycles. Therefore, the electrochromic switching behavior of PAs appears to be a highly reversible process. In contrast, PI **7a** exhibited a higher oxidation potential and significantly decreased the cycling stability than the corresponding PA **5b** analog. The result can be explained by its lowered electrochemical stability arising from the electron-withdrawing effect of the imide units.

The percentage transmittance change (Δ%*T*) between neutral (at 0.0 V) and oxidized (at 1.05 V) states of the PA **5b** film was up to 84% at 828 nm. PA **5b** attained 90% of a complete blue-green coloring in 3.77 s, as shown in Figure S17 (Supplementary Materials). In contrast, PI **7a** attained 90% oxidized blue states in 4.33 s. The electrochromic coloration efficiency (CE) value of PA **5b** was found to be 186 cm^2^ C^−1^ at 828 nm by chronoamperometry. PI **7a** showed a slightly lower CE value of 133 cm^2^ C^−1^ at 754 nm.

### 3.5. Electrochromic Devices

**Figure 11:**
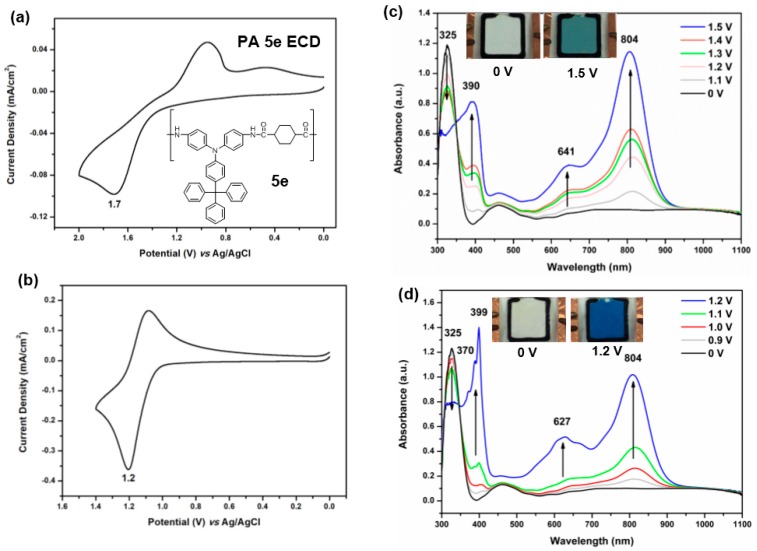
(**a**) Cyclic voltammetry (CV) diagram of the Electrochromic Devices (ECD) (ITO/PA **5e** film/gel electrolyte/ITO) based on the cast film (thickness: 250 ± 30 nm) of PA **5e** on the ITO-coated glass substrate; (**b**) CV diagram of the ECD based on PA **5e**/HV; (**c**) Spectra and color change of the ECD based on PA **5e** at indicated applied voltages; (**d**) Spectra and color change of the PA **5e**/HV ECD at indicated applied voltages. The semi-gelled electrolyte consists of 10-wt % PMMA (average *M*w = 120,000) in 0.6 M LiBF_4_/propylene carbonate.

**Figure 12:**
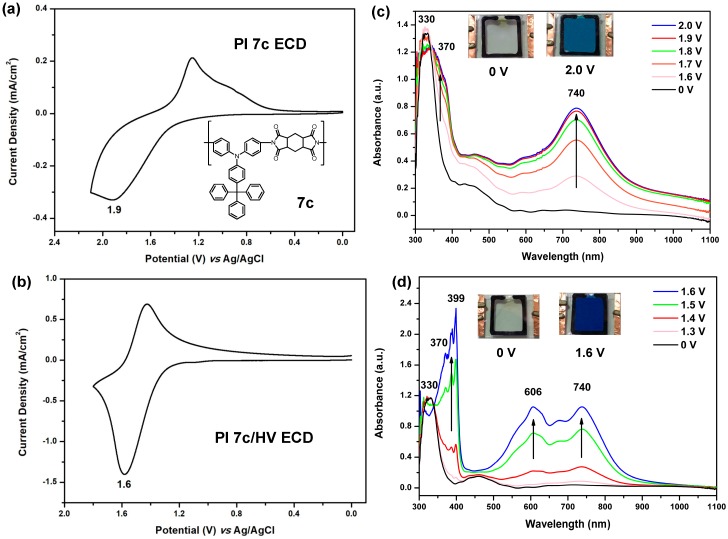
(**a**) CV diagram of the ECD (ITO/PI **7c** film/gel electrolyte/ITO) based on the cast film (thickness: 250 ± 30 nm) of PI **7c** on the ITO-coated glass substrate; (**b**) CV diagram of the ECD based on PI **7c**/HV; (**c**) Spectra and color change of the ECD based on PI **7c** at indicated applied voltages; (**d**) Spectra and color change of the PI **7c**/HV ECD at indicated applied voltages. The semi-gelled electrolyte consists of 10-wt % PMMA (average *M*w = 120,000) in 0.6 M LiBF_4_/propylene carbonate.

The semi-aromatic PA **5e** and PI **7c** films were chosen as active layers in the fabication of ECDs due to the fact that they are almost colorless in neutral state and are expected to exhibit high electrochromic contrast upon oxidation. The CV diagrams and electrospectrographs of **5e** and **5e/**HV devices are illustrated in Figure 11. As shown in Figure 11a, the **5e** device revealed one oxidation peak at 1.7 V. The high working potential might result in waste of energy and less stability of the devices. In order to improve these drawbacks, the cathodically electrochromic material HV was introduced into the semi-gelled electrolyte layer of ECDs. The CV diagram of **5e**/**HV** is depicted in Figure 11b, demonstrating reversible redox steps with a decreased oxidative potential (*E*_pa_ at 1.2 V). This effect could be ascribed to HV^2+^ having the capability to accept electrons from TPA moieties during the oxidation process, and then the resulted HV^+^ also could donate electrons back to TPA radical cations during the reducing process. The spectroelectrochemical graphs of **5e** ECD are presented in Figure 11c. The spectra and color changes are similar to those observed in the electrochemical cell. Spectroelectrochemical behavior of ECDs **5e**/HV was also studied and depicted in Figure 8d. New absorption peaks could be observed at 366 nm and 627 nm due to the reductive process of HV^+2^ to HV^+^. Because of the combination of TPA oxidation and HV reduction at the same time, the dual ECD revealed a deeper blue coloring as the potential was applied. Similar results were also observed for the **7c** and **7c**/HV ECDs as shown in Figure 12. Thus, by adding HV as an efficient charge trapping agent in electrolyte layer, not only the working voltage could be reduced but also the performance of overall system was enhanced.

## 4. Conclusions

Electroactive polyamides and polyimides containing trityl-triphenylamine units were successfully synthesized and characterized. Due to the bulky, double propeller-shaped structure of the diamine component, all of the prepared polymers exhibit high solubility in various organic solvents and could afford flexible or robust films. The polymers derived from aliphatic dicarboxylic acid or dianhydride could afford almost colorless films. The wholly aromatic polyamides and polyimides exhibited good thermal stability and could afford flexible and strong films. All of the cast films on the ITO electrode revealed reversible redox behavior upon electro-oxidation, accompanying with a green-blue or blue coloring change. The polyimides exhibited a slightly higher oxidation potential and less stable cycling stability than the corresponding polyamide analogs. The electrochromic devices using the prepared polymers as active layers showed promising performance. Additionally, incorporating HV into the electrolyte as charge balance material led to a decreased working potential and an increased contrast in electrochromic coloring.

## Figures and Tables

**Table 1:** Inherent viscosity and organosolubility of the polymers.

Polymer Code	η_inh_ (dL g^−1^) ^a^	Solubility in Various Solvents ^b,c^					
		NMP	DMAc	DMF	DMSO	*m*-Cresol	THF
**5a**	0.49	++	++	++	++	++	+−
**5b**	0.62	++	++	++	++	++	+−
**5c**	0.43	++	++	++	++	++	++
**5d**	0.42	++	++	++	++	++	++
**5e**	0.35	++	++	++	++	++	++
**7a**	0.25	++	++	+	+	++	++
**7b**	0.64	++	++	++	+	++	++
**7c**	0.30	++	++	++	++	++	++

^a^ Inherent viscosity of the polymer solution at a concentration of 0.5 g dL^−1^ in DMAc at 30 °C; ^b^ NMP: *N*-methyl-2-pyrrolidone; DMAc: *N*,*N*-dimethylacetamide; DMF: *N*,*N*-dimethylformamide; DMSO: dimethyl sulfoxide; THF: tetrahydrofuran; ^c^ The solubility was determined using 10 mg of the polymer sample and 1 mL of solvent. ++, soluble at room temperature; +, soluble on heating; +−, partially soluble on heating.

**Table 2:** Thermal properties of the polymers.

Polymer	*T*_g_ (°C)	*T*_d_^10^ (°C)		Char Yield at 800 °C (wt %)
		in N_2_	in Air	
**5a**	312	410	420	55
**5b**	288	430	430	55
**5c**	305	435	460	55
**5d**	206	400	400	27
**5e**	295	395	390	32
**7a**	292	570	560	60
**7b**	314	570	550	67
**7c**	336	525	520	39

**Table 3:** Electrochemical properties of PAs **5a**–**5e** and PIs **7a**–**7c**.

Polymer	UV-Vis Absorption (nm) ^a^		Oxidation Potential (V) ^b^		*E*_g_ (eV) ^c^	HOMO (eV) ^d^	LUMO (eV) ^d^
	λ_max_	λ_onset_	*E*_onset_	*E*_1/2_^Ox^			
**5a**	352	436	0.70	0.85	2.84	−5.51	−2.67
**5b**	340	396	0.74	0.85	3.13	−5.51	−2.38
**5c**	343	414	0.72	0.86	2.99	−5.52	−2.53
**5d**	320	368	0.70	0.83	3.37	−5.49	−2.12
**5e**	321	373	0.71	0.81	3.32	−5.47	−2.15
**7a**	320	379	1.02	1.11	3.27	−5.77	−2.50
**7b**	323	379	1.00	1.10	3.27	−5.76	−2.49
**7c**	316	358	0.99	1.09	3.46	−5.75	−2.29

^a^ Measured as solid thin films; ^b^ Obtained from CV diagrams, relative to Ag/AgCl in 0.1 M TBAP/MeCN and the scan rate was 50 mV s^−1^; ^c^ Optical bandgap calculated from the UV–vis absorption edge of the polymer film: *E*_g_ = 1240/λ_onset_; ^d^ *E*_HOMO_ = −[*E*_1/2_^Ox^ + 5.1 − 0.44] (eV); *E*_LUMO_ = −(*E*_HOMO_ + *E*_g_).

**Table 4:** Coloration efficiency of polyamide **5b**.

Cycling Times ^a^	ΔOD_490_^b^	Q_d_ (mC cm^−2^) ^c^	CE (cm^2^ C^−1^) ^d^	Decay in CE (%)
1	0.99	5.33	186	0
10	0.99	5.33	186	0
20	0.98	5.31	185	0.5
30	0.98	5.31	185	0.5
40	0.98	5.32	184	1
50	0.97	5.30	183	1.6
60	0.96	5.29	181	2.7
70	0.96	5.30	181	2.7
80	0.96	5.29	180	3.2
90	0.95	5.30	179	3.7
100	0.94	5.28	178	4.3

^a^ Applied potential was switched between 0 and 1.05 V (vs. Ag/AgCl); ^b^ Optical Density (ΔOD) = log[T_bleached_/T_colored_], where T_colored_ and T_bleached_ are the maximum transmittance in the oxidized and neutral states, respectively; ^c^ Ejected charge, obtained from the in situ experiments; ^d^ Coloration efficiency calculated by CE = ΔOD/Q_d_.

## Supplementary Materials

The following are available online at www.mdpi.com/2073-4360/9/10/511/s1, Figure S1: IR spectra of compounds **1** to **3**; Figure S2: ^1^H NMR spectra of compounds **1** and **2** in DMSO-*d*_6_; Figure S3: Mass analysis of compounds **2** and **3**; Figure S4: IR spectra of PAs **5a**−**5e**; Figure S5: ^1^H H-H COSY NMR spectra of polyamide **5a** in DMSO-*d*_6_; Figure S6: IR spectra of PIs **7a**−**7c**; Figure S7: ^1^H H-H COSY NMR spectra of polyimide **7b** in DMSO-*d*_6_; Figure S8: TGA curves of PAs **5c** and **5d** and PIs **7a**−**7c**; Figure S9: Cyclic voltammogram of 1 mM ferrocene in 0.1 M Bu_4_NClO_4_/MeCN at a scan rate of 50 mV·s^−1^; Figure S10: Cyclic voltammograms of PAs **5b**–**5e** films on the ITO-coated glass substrate in 0.1 M Bu_4_NClO_4_/MeCN at a scan rate of 50 mV·s^−1^; Figure S11: Cyclic voltammograms of PIs **7b** and **7c** films on the ITO-coated glass substrate in 0.1 M Bu_4_NClO_4_/MeCN at a scan rate of 50 mV·s^−1^; Figures S12–S16: Optical absorption spectra of PAs and PIs. Figure S17: Current monitored and optical transmittance changes of the PA **5b** and PI **7a** films while the potential was switched.
