# Supplementary material for: Synthesis and Electrochromism of Highly Organosoluble Polyamides and Polyimides with Bulky Trityl-Substituted Triphenylamine Units
Source: Polymers (Basel). 2017 Oct 14;9(10):511. doi: 10.3390/polym9100511 (PMC6419009; doi:10.3390/polym9100511)
Supplement: Supplementary file 1 [file polymers-09-00511-s001.docx]

**Supplementary Materials**

**Synthesis and Electrochromism of Highly Organosoluble Polyamides and Polyimides with Bulky Trityl-substituted Triphenylamine Units**

**Sheng-Huei Hsiao^1*^, Wei-Kai Liao^1,2^, Guey-Sheng Liou^2^ ***

^1^ Department of Chemical Engineering and Biotechnology, National Taipei University of Technology, No. 1, Sec. 3, Chunghsiao East Rd., Taipei 10608, Taiwan; [s880497@gmail.com](mailto:s880497@gmail.com) (W.-K. Liao)

^2^ Institute of Polymer Science and Engineering, National Taiwan University, No. 1, Sec. 4, Roosevelt Rd., Taipei 10617, Taiwan

*Correspondence: shhsiao@ntut.edu.tw; Tel.: +886-2-27712171 ext. 2548; Fax: +886-2-27317117 or [gsliou@ntu.edu.tw](mailto:gsliou@ntu.edu.tw); Tel.: +886-2-33665315; Fax: +886-2-33665237

***Instrumentation and Measurements***

Infrared (IR) spectra of the synthesized compounds (KBr pellets) and polymers (films) were obtained on a Horiba FT-720 FT-IR spectrometer. NMR spectra were recorded on a Bruker Avance III HD-600 MHz NMR spectrometer (^1^H and ^13^C, 150 MHz and 600 MHz respectively). Elemental analyses were run in a Heraeus Vario EL III CHNS elemental analyzer. HRMS were obtained on a JEOL JMS-700 mass spectrometer with ESI resource. The inherent viscosities were determined with a Cannon-Fenske viscometer at 30 ^o^C. Thermogravimetric analysis (TGA) was performed with a Perkin-Elmer Pyris 1 TGA instrument. The samples were heated at 300 ^o^C for 1 h prior to all the thermal analyses. TGA experiments were carried out on approximately 4−6 mg of film samples heated in flowing nitrogen or air (flow rate = 20 cm^3^ min^−1^) at a heating rate of 20 ^o^C min^−1^. DSC analyses were performed on a Perkin-Elmer DSC 4000 differential scanning calorimeter at a scan rate of 20 ^o^C min^−1^ in flowing nitrogen. The samples were heated from 50 to 400 ^o^C at a scan rate of 20 ^o^C min^−1^ followed by rapid cooling to 50 ^o^C at −200 ^o^C min^−1^ in nitrogen. The midpoint temperature of baseline shift on the subsequent DSC trace (from 50 ^o^C to 400 ^o^C at a heating rate 20 ^o^C min^−1^) was defined as *T*_g_. Electrochemistry was performed with a CHI 750A electrochemical analyzer. Cyclic voltammetry was conducted with the use of a three-electrode cell in which ITO (polymer film area about 0.8 cm x 1.25 cm) was used as a working electrode. A platinum wire was used as an auxiliary electrode. All cell potentials were taken with the use of a home-made Ag/AgCl, KCl (*sat.*) reference electrode. Ferrocene was used as an external reference for calibration (+0.48 V vs. Ag/AgCl). Voltammograms are presented with the positive potential pointing to the left and with increasing anodic currents pointing downwards. Spectroelectrochemistry analyses were carried out with an electrolytic cell, which was composed of a 1 cm cuvette, ITO as a working electrode, a platinum wire as an auxiliary electrode, and a Ag/AgCl reference electrode. Absorption spectra in the spectroelectrochemical experiments were measured with an Agilent 8453 UV-Visible diode array spectrophotometer. Color coordinates of the electrochromic films were measured on an Admesy Brontes colorimeter. Thickness of polymer films was measured with a surface profiler (Kosaka Lab., Surfcorder ET3000, Japan).


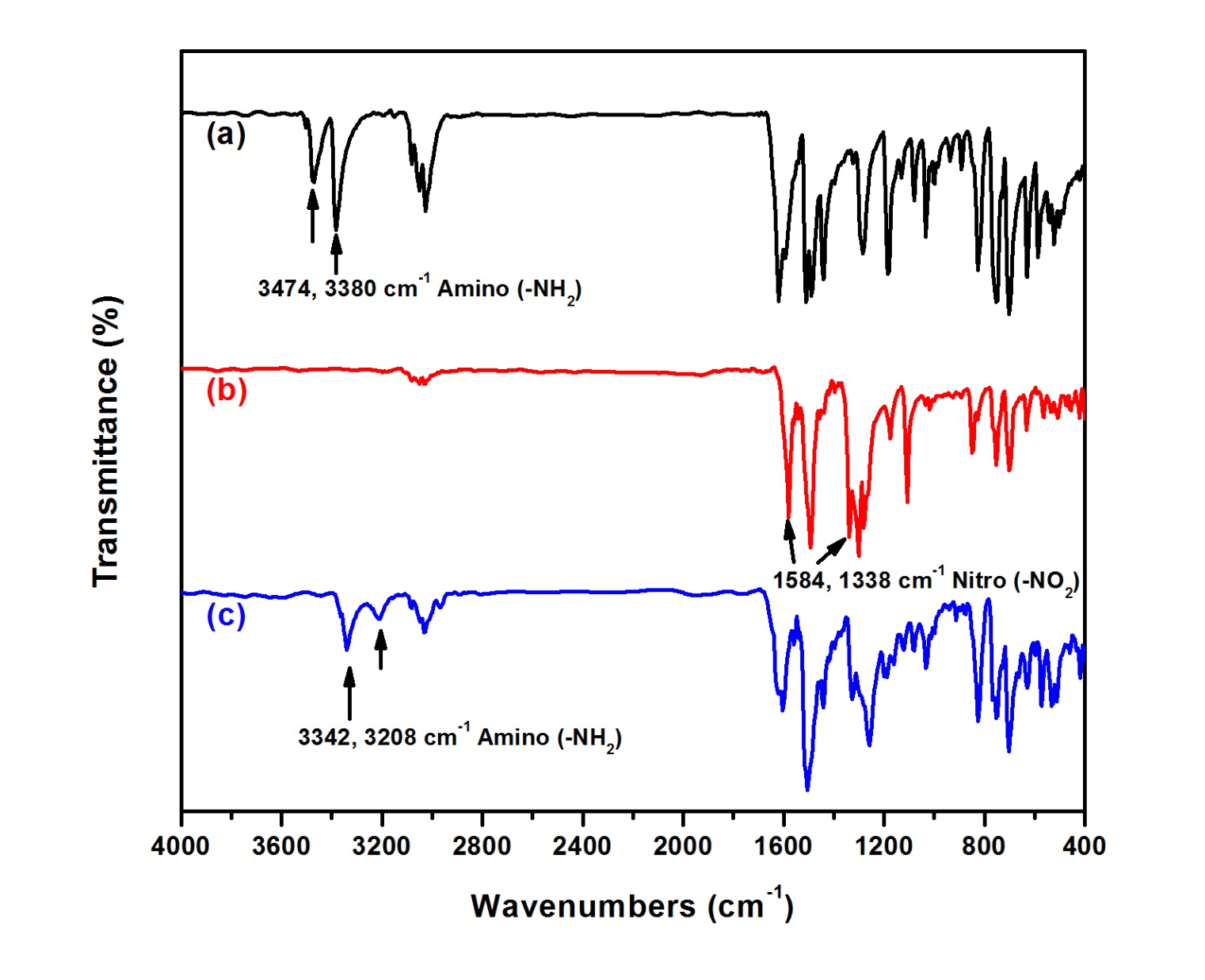

Figure S1. IR spectra of compounds (a) 1, (b) 2 and (c) 3.


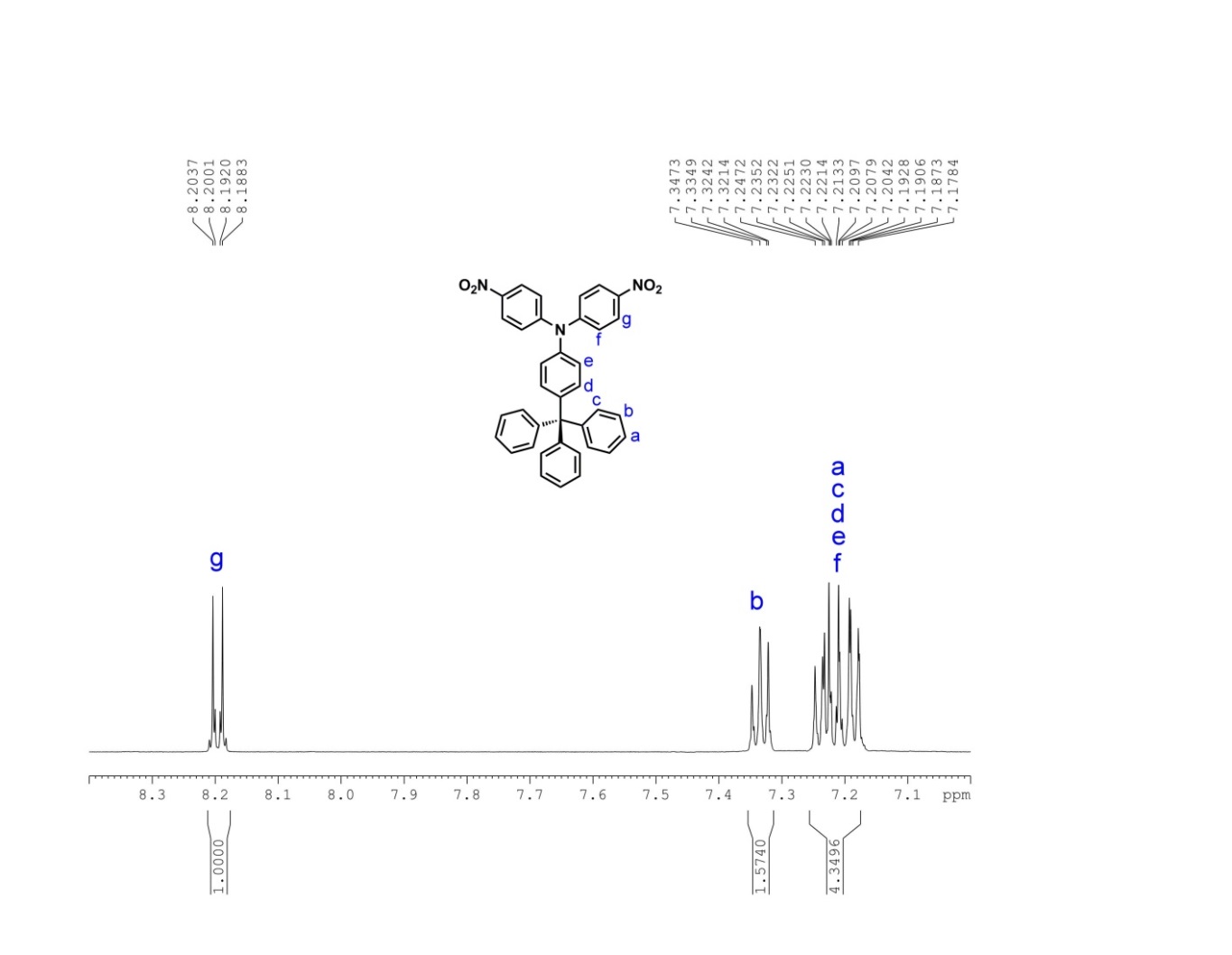

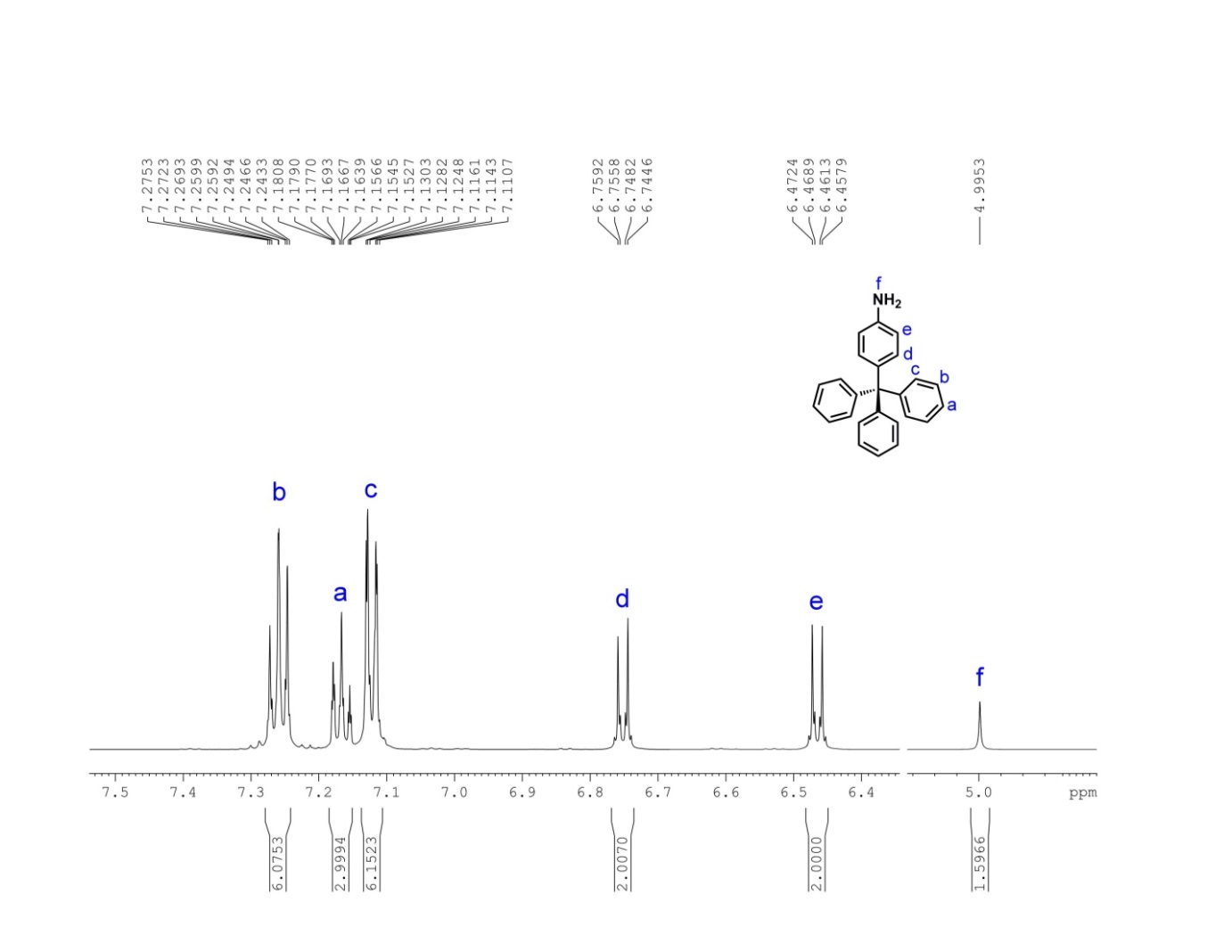


**Figure S2.** ^1^H NMR spectra of (a) 4-tritylaniline (**1)** and (b) dinitro compound **2** in DMSO-*d_6_*.

**Figure S3.** Mass spectra of compounds **2** and **3**.





**Figure S4.** IR spectra of polyamides **5a−5e**.


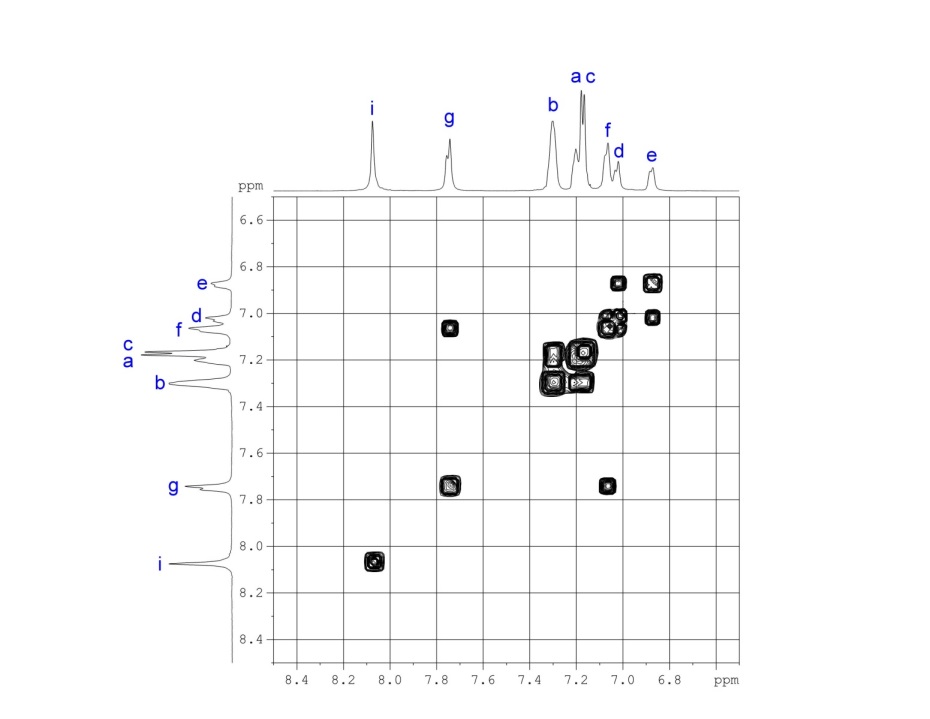

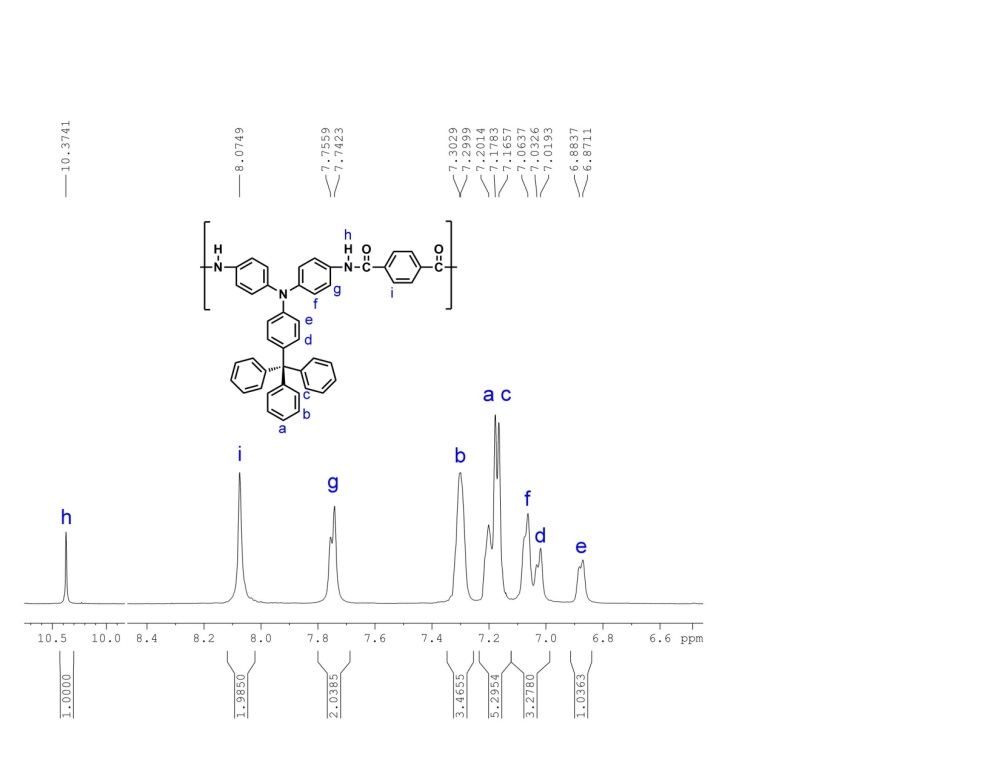


**Figure S5.** ^1^H and H-H COSY NMR spectra of polyamide **5a** in DMSO-*d*_6_.

**

**

**Figure S6.** IR spectra of polyimides **7a−7c**.


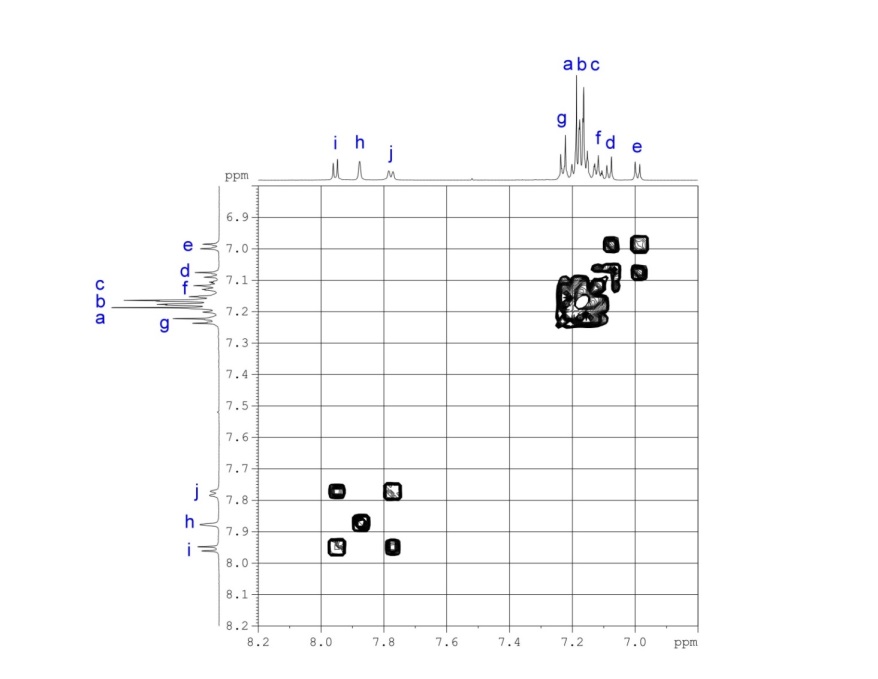

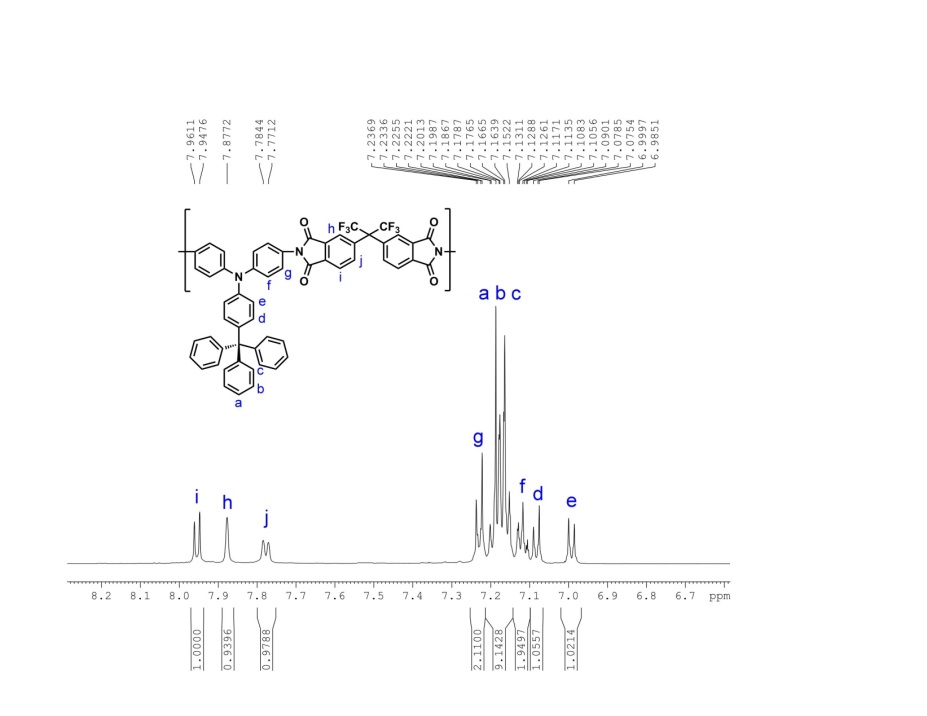


**Figure S7.** ^1^H and H-H COSY NMR spectra of polyimide **7b** in DMSO-*d*_6_.












**Figure S8.** TGA curves of PAs **5c** and 5d and PIs **7a−7c** with a heating rate of 20 ^o^C min^−1^ in nitrogen and air atomspheres.





**Figure S9.** Cyclic voltammogram of 1 mM ferrocene in 0.1 M Bu_4_NClO_4_/MeCN at a scan rate of 50 mV s^−1^.












**Figure S10.** Cyclic voltammograms of PAs **5b−5e** films on the ITO-coated glass substrate in 0.1 M Bu_4_NClO_4_/MeCN at a scan rate of 50 mV s^−1^.







**Figure S11.** Cyclic voltammograms of PIs **7b** and **7c** films on the ITO-coated glass substrate in 0.1 M Bu_4_NClO_4_/MeCN at a scan rate of 50 mV s^−1^.

**L*= 70**

**a*= −4**

**b*= 11**

**L*= 58**

**a*= −15**

**b*= 14**


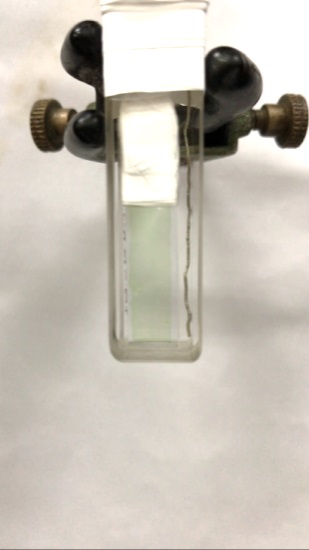

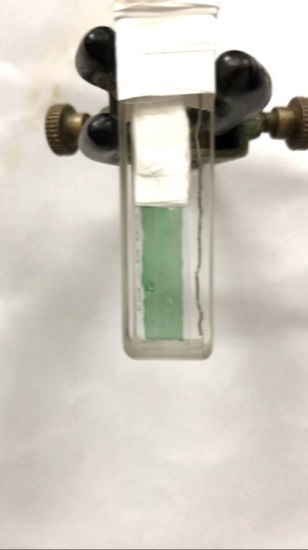


**0 V**

**1.0 V**


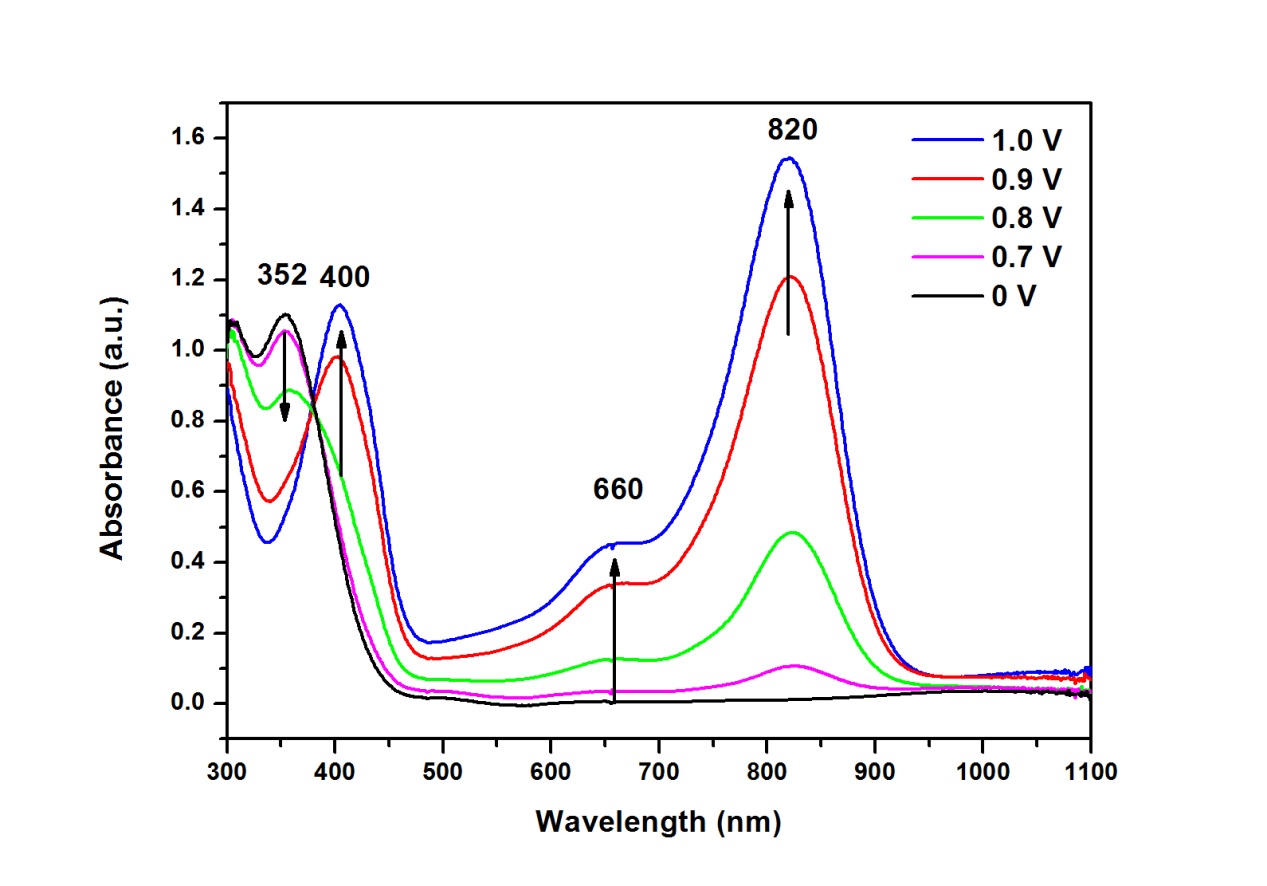


**Figure S12.** Optical absorption spectra of the cast film of polyamide **5a** (thickness: 200 ± 30 nm) on the ITO-coated glass substrate in 0.1 M Bu_4_NClO_4_/MeCN at various applied potentials between 0.0 V and 1.0 V.

**L*= 72**

**a*= 0**

**b*= 3**

**L*= 52**

**a*= −10**

**b*= 2**


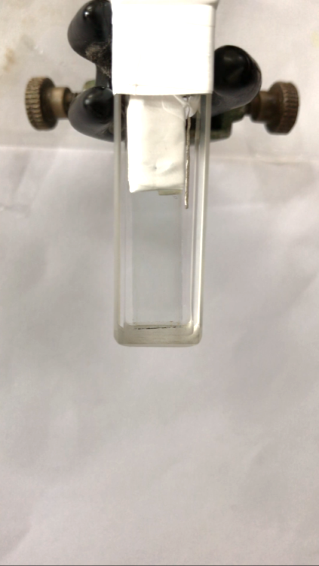

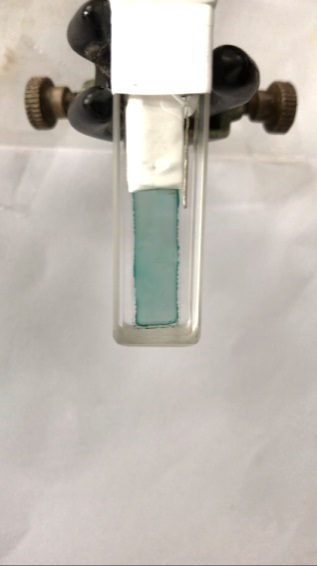


**0 V**

**1.0 V**


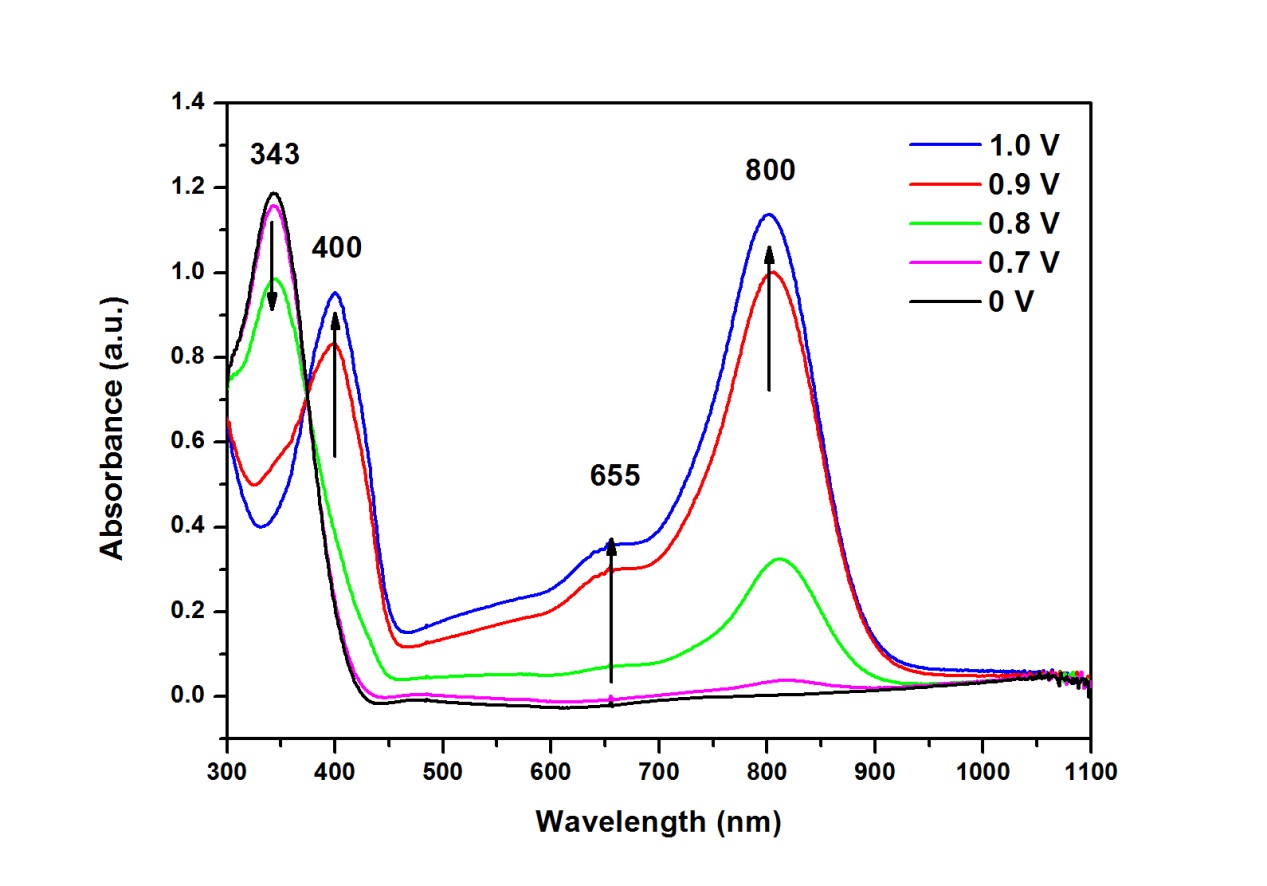


**Figure S13.** Optical absorption spectra of the cast film of polyamide **5c** (thickness: 200 ± 30 nm) on the ITO-coated glass substrate in 0.1 M Bu_4_NClO_4_/MeCN at various applied potentials between 0.0 V and 1.0 V.

**L*= 51**

**a*= −9**

**b*= −5**

**L*= 68**

**a*= 1**

**b*= 3**

**L*= 56**

**a*= −4**

**b*= −14**

**L*= 70**

**a*= −1**

**b*= 2**

**L*= 71**

**a*= −1**

**b*= 7**

**(a)**

**(b)**


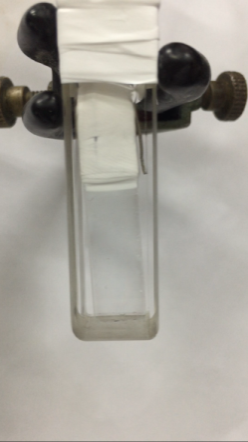

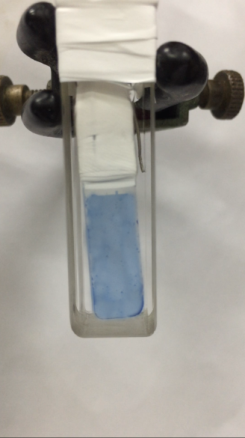


**0 V**

**1.0 V**


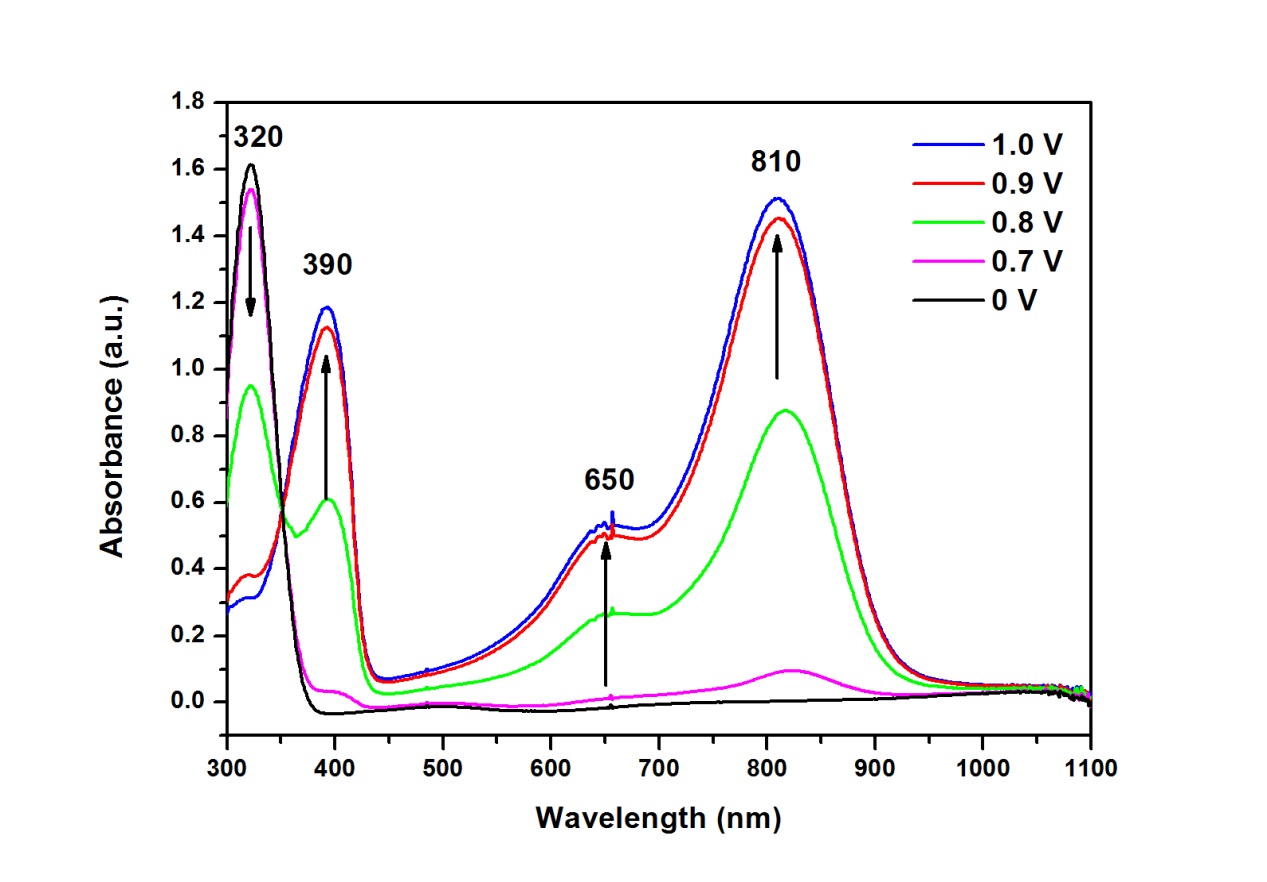

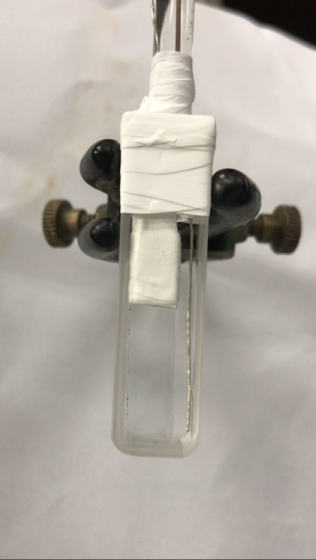

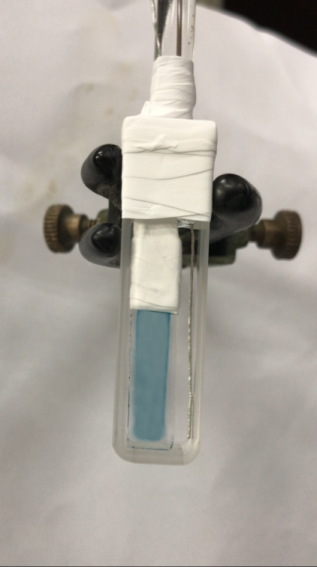


**0 V**

**1.0 V**


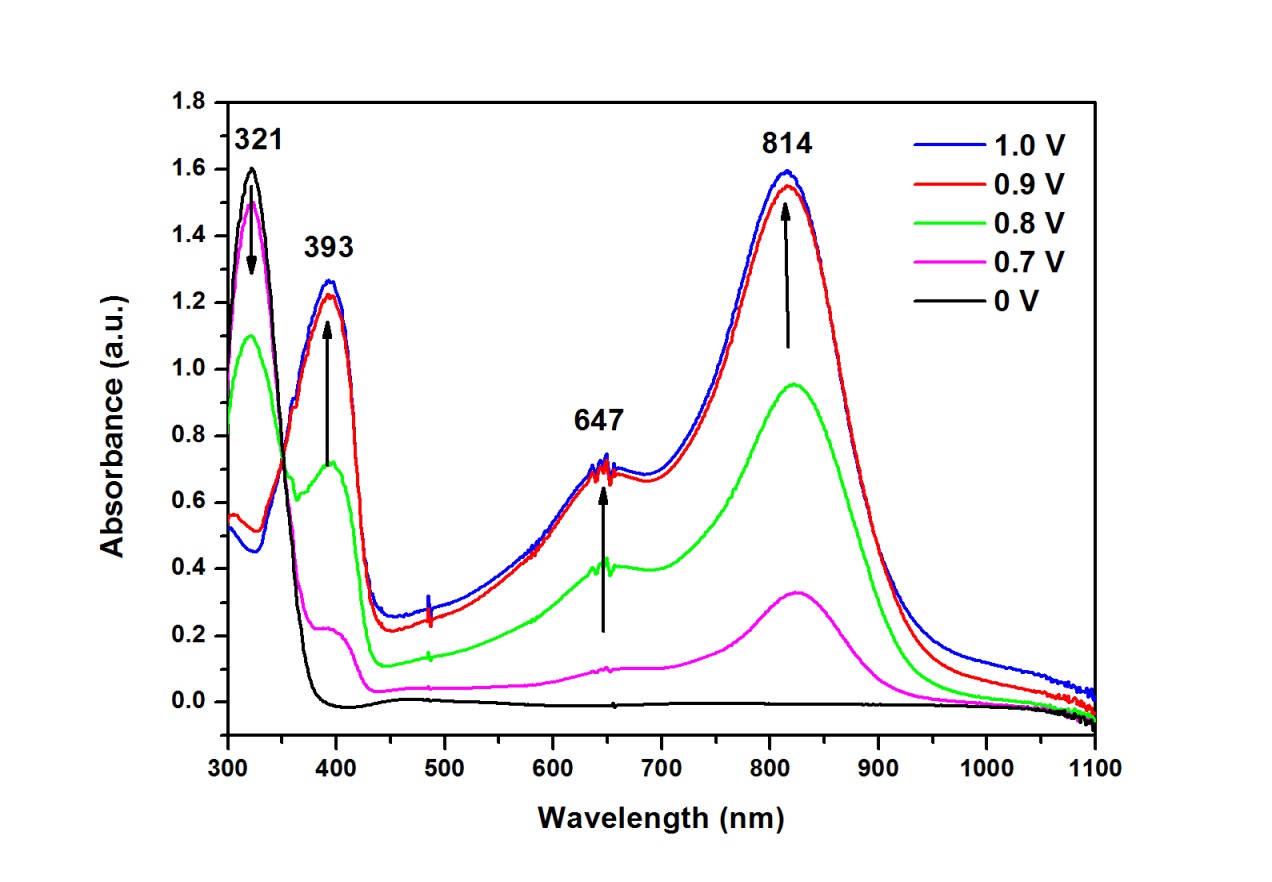


**Figure S14.** Optical absorption spectra of the cast films of polyamides (a) **5d** (thickness: 200 ± 30 nm) and (b) **4e** (thickness: 200 ± 30 nm) on the ITO-coated glass substrate in 0.1 M Bu_4_NClO_4_/MeCN at various applied potentials between 0.0 V and 1.0 V.

**
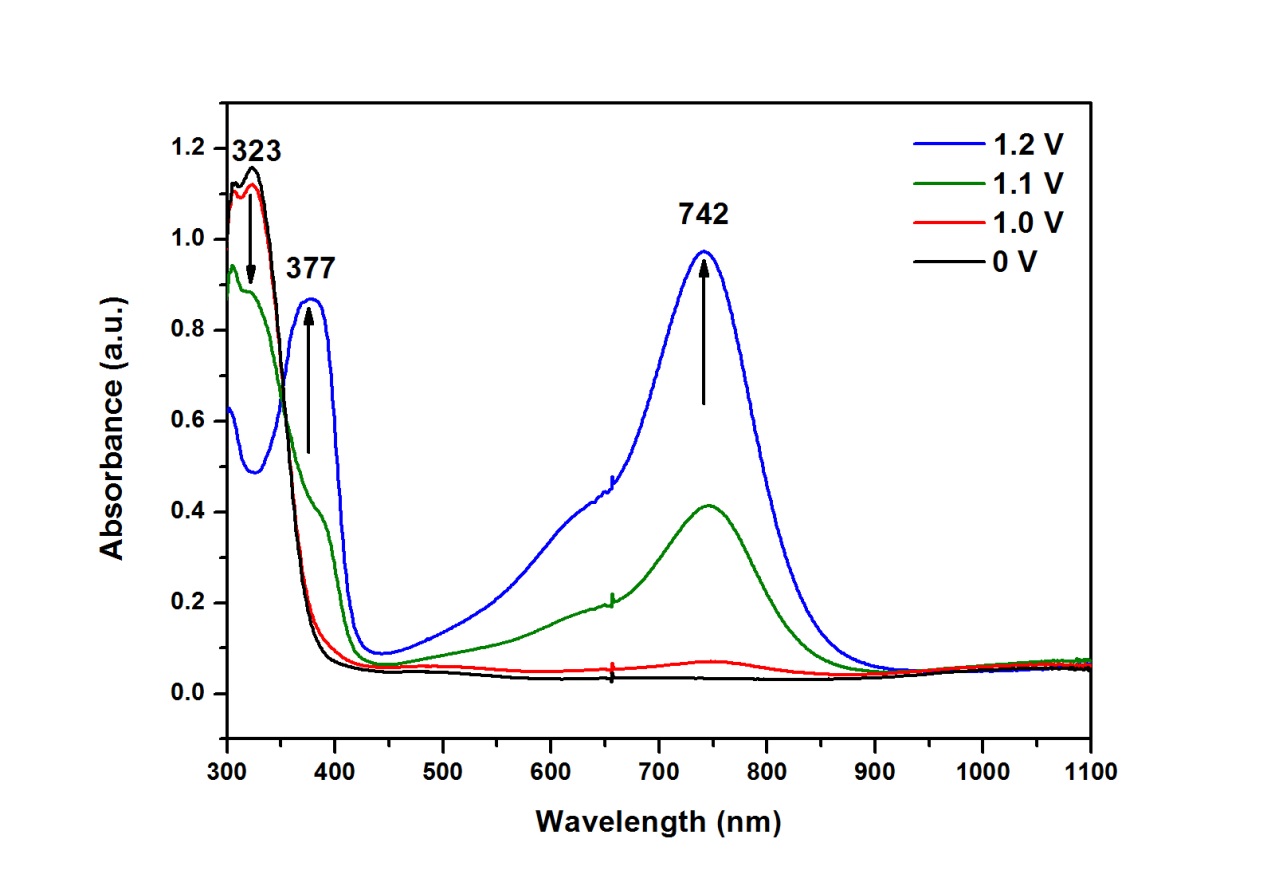
**

**L*= 55**

**a*= −7**

**b*= -7**

**L*= 66**

**a*= −1**

**b*= 5**

**0 V**

**1.2 V**


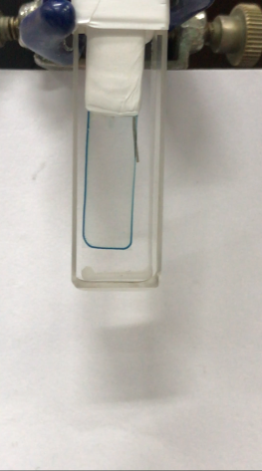

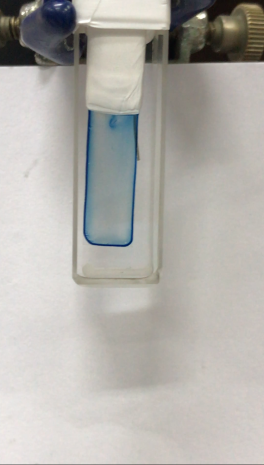


**Figure S15.** Optical absorption spectra of the cast film of polyimide **7b** (thickness: 200 ± 30 nm) on the ITO-coated glass substrate in 0.1 M Bu_4_NClO_4_/MeCN at various applied potentials.

**
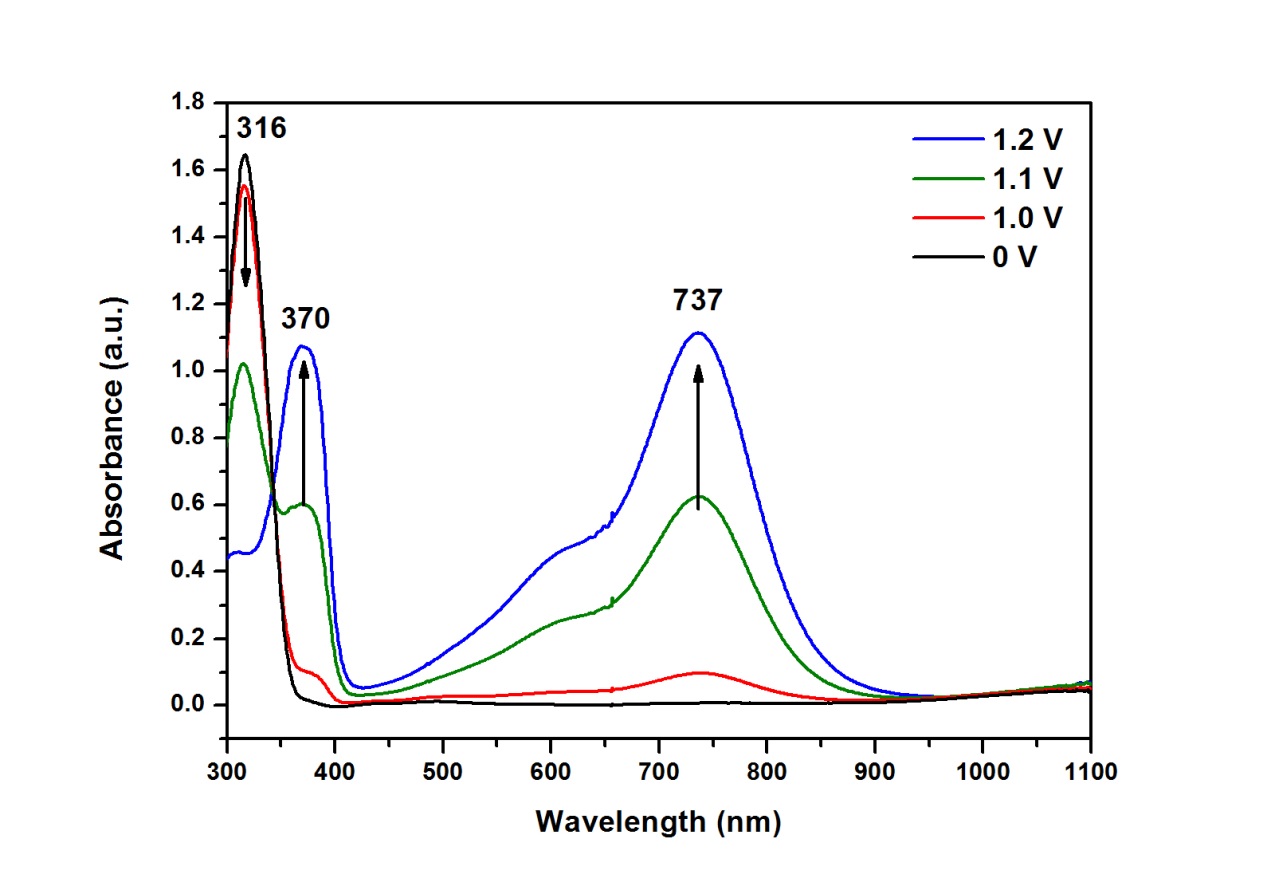
**

**0 V**

**1.2 V**


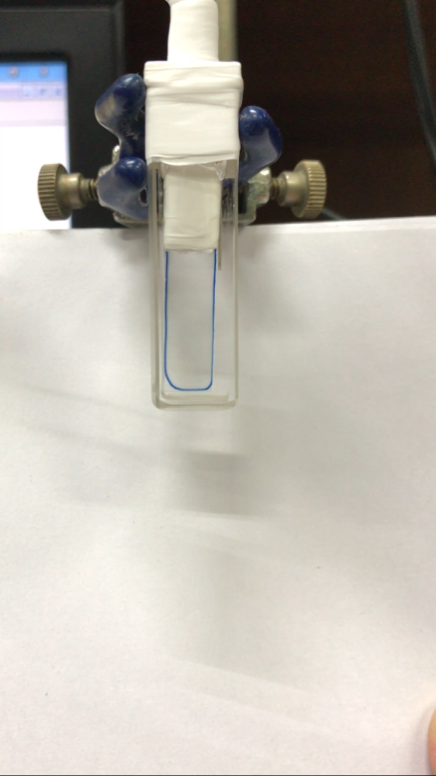

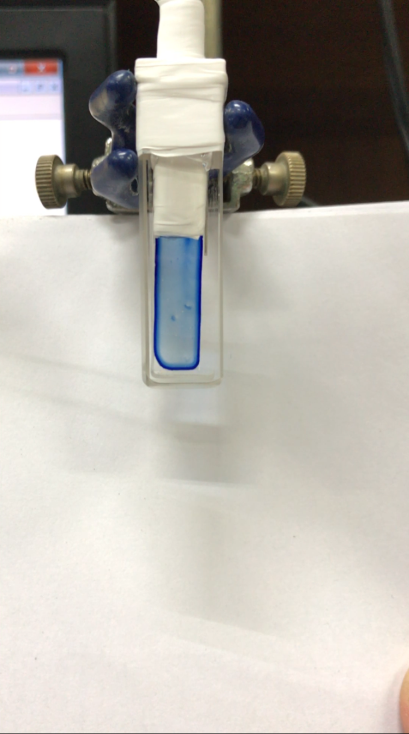


**L*= 51**

**a*= −7**

**b*= −9**

**L*= 71**

**a*= −1**

**b*= 7**

**Figure S16.** Optical absorption spectra of the cast film of polyimide **7c** (thickness: 200 ± 30 nm) on the ITO-coated glass substrate in 0.1 M Bu_4_NClO_4_/CH_3_CN at at various applied potentials.

**(b)**

**(a)**


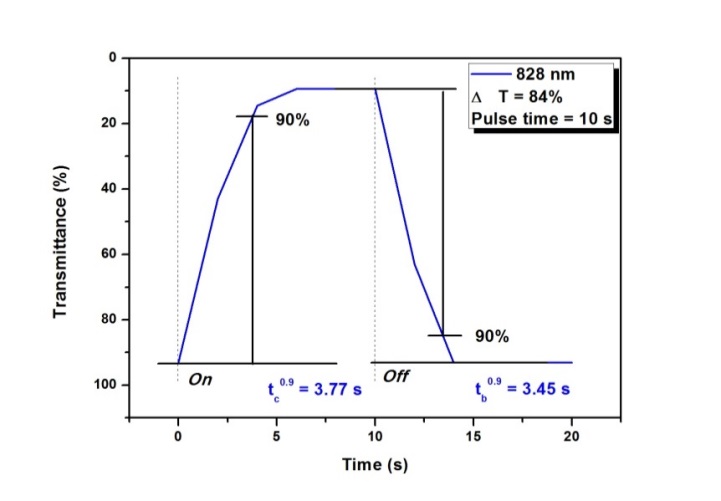

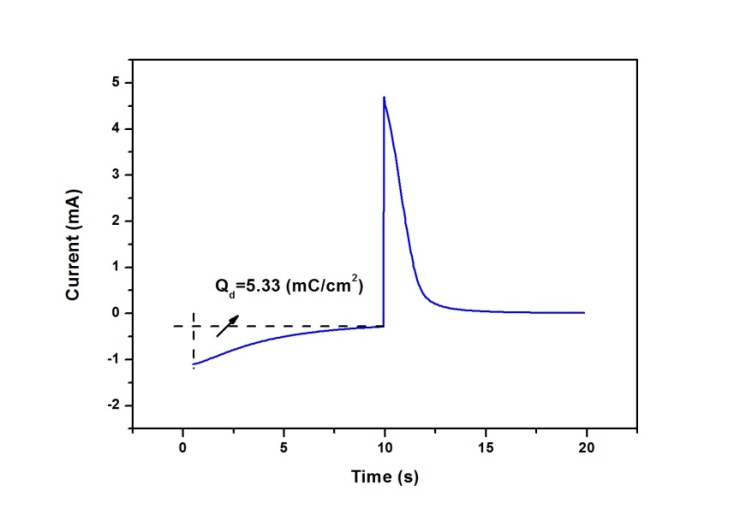

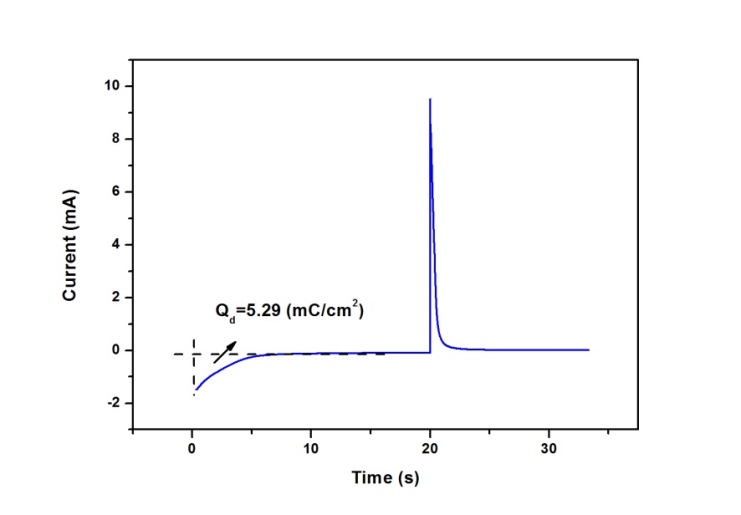

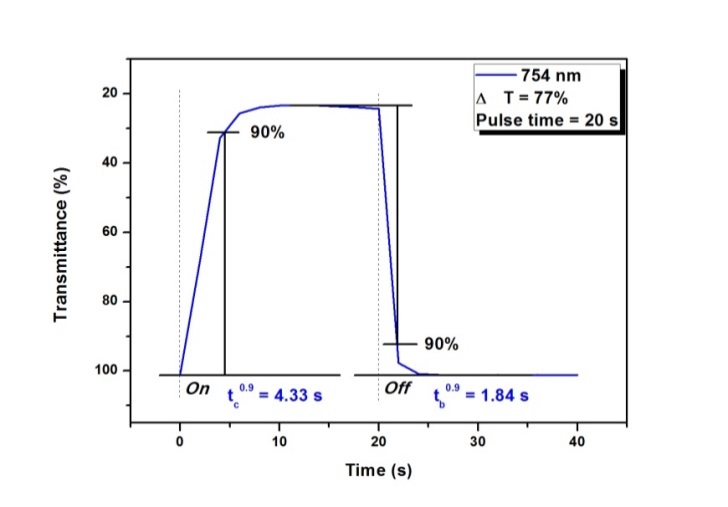


**Figure S17.** Current monitored and optical transmittance changes of the film (thickness: 200 ± 30 nm) on the ITO-glass slide in 0.1 M Bu_4_NClO_4_/CH_3_CN while the potential was switched: (a) PA **5b** film between 0.0 V and 1.05 V at 828 nm with a pulse time of 10 s and (b) PI **7a** film between 0.0 V and 1.30 V at 754 nm with a pulse time of 20 s.
